# Endovascular Treatment with Stent-Retriever Devices for Acute Ischemic Stroke: A Meta-Analysis of Randomized Controlled Trials

**DOI:** 10.1371/journal.pone.0147287

**Published:** 2016-01-25

**Authors:** Chad K. Bush, Dayaamayi Kurimella, Lee J. S. Cross, Katherine R. Conner, Sheryl Martin-Schild, Jiang He, Changwei Li, Jing Chen, Tanika Kelly

**Affiliations:** 1 Department of Epidemiology, Tulane University School of Public Health and Tropical Medicine, New Orleans, Louisiana, United States of America; 2 Department of Medicine, Tulane University School of Medicine, New Orleans, Louisiana, United States of America; 3 Department of Neurology, Tulane University School of Medicine, New Orleans, Louisiana, United States of America; National Cerebral and Cardiovascular Center, JAPAN

## Abstract

**Importance:**

Acute ischemic stroke is a leading cause of death and disability worldwide. Several recent clinical trials have shown that endovascular treatment improves clinical outcomes among patients with acute ischemic stroke.

**Objective:**

To provide an overall and precise estimate of the efficacy of endovascular treatment predominantly using second-generation mechanical thrombectomy devices (stent-retriever devices) compared to medical management on clinical and functional outcomes among patients with acute ischemic stroke.

**Data Sources:**

MEDLINE, EMBASE, Cochrane Collaboration Central Register of Controlled Clinical Trials, Web of Science, and NIH ClinicalTrials.gov were searched through November 2015.

**Study Selection:**

Searches returned 3,045 articles. After removal of duplicates, two authors independently screened titles and abstracts to assess eligibility of 2,495 potentially relevant publications. From these, 38 full-text publications were more closely assessed. Finally, 5 randomized controlled trials of endovascular treatment with predominant use of retrievable stents were selected.

**Data Extraction and Synthesis:**

Three authors independently extracted information on participant and trial characteristics and clinical events using a standardized protocol. Random effects models were used to pool endovascular treatment effects across outcomes.

**Main Outcomes and Measures:**

The primary outcome was better functional outcome as measured on the modified Rankin Scale at 90 days of follow-up. Secondary outcomes included all-cause mortality and symptomatic intra-cerebral hemorrhage.

**Results:**

Five trials representing 1,287 patients were included. Overall, patients randomized to endovascular therapy experienced 2.22 times greater odds of better functional outcome compared to those randomized to medical management (95% CI, 1.66 to 2.98; P < 0.0001). Endovascular therapy was not associated with mortality [OR (95% CI), 0.78 (0.54, 1.12); P = 0.1056] or symptomatic intracerebral hemorrhage [OR (95% CI), 1.19 (0.69, 2.05); P = 0.5348]. Meta-regression analysis suggested that shorter times from stroke onset to groin puncture and from stroke onset to reperfusion result in better functional outcomes in ischemic stroke patients (P = 0.0077 and P = 0.0089). There were no significant differences in the beneficial effects of endovascular treatment on functional outcomes across categories of gender, age, stroke severity, ischemic changes on computed tomography, or intravenous tissue plasminogen activator administration.

**Conclusions and Relevance:**

This meta-analysis demonstrated superior functional outcomes in subjects receiving endovascular treatment compared to medical management. Further, this analysis showed that acute ischemic stroke patients may receive enhanced functional benefit from earlier endovascular treatment.

## Introduction

Stroke is a leading cause of morbidity and mortality worldwide [1, 2]. In the US, 87% of all strokes are ischemic [2], caused by occlusion of extra- or intra-cranial cerebral arteries by thrombus or embolism, leading to necrosis and cell death of brain tissue and neurologic deficits indicative of the affected area. Thrombolytic therapy with intravenous tissue plasminogen activator (IV t-PA) has been recommended as the standard treatment for acute ischemic stroke when a patient qualifies [3]. It has been shown that IV t-PA is clinically effective within 4.5 hours after onset of stroke symptoms, after which the likelihood of neurological and functional recovery decreases [4]. Because of the short therapeutic window for IV t-PA, and because of the extensive set of other clinical eligibility criteria for administration, limited acute ischemic stroke patients qualify for the intervention on presentation [5].

Endovascular therapy, involving minimally invasive techniques for intra-arterial thrombolysis or mechanical thrombectomy, has been explored as an alternative or adjunct to medical management for many years [6, 7]. Although endovascular therapy had been associated with a higher probability of recanalization [7], results of three 2013 trials (IMS III [8], SYNTHESIS Expansion [9], and MR RESCUE [10]) showed no significant benefit of endovascular treatment predominantly using first-generation mechanical thrombectomy devices compared to medical management on stroke outcomes [8–10]. The results and effect estimates from these trials were subsequently summarized and pooled in meta-analyses, at the time supporting the findings of no benefit for endovascular treatment compared to IV thrombolysis alone [11, 12].

In contrast, recently published trials (MR CLEAN [13], ESCAPE [14], EXTEND-IA [15], SWIFT PRIME [16], and REVASCAT [17]) have demonstrated significant benefits of endovascular therapy with second-generation mechanical thrombectomy devices (stent-retrievers) in acute ischemic stroke patients [13–17]. Given the conflicting results between the trials published in 2013 and the most recent results, we performed a meta-analysis of randomized controlled trials examining the effects of endovascular treatment with second-generation mechanical thrombectomy devices in order to explain discrepancies and to provide an overall and precise estimate of treatment effects which may be used to guide clinical practice and policy development.

The aim of our study was to perform a meta-analysis of all published randomized controlled trials that compare the efficacy of endovascular treatment predominantly using second-generation mechanical thrombectomy devices to medical management alone in patients with acute ischemic stroke. Our primary outcome was the modified Rankin Scale (mRS) score as a measure of the degree of disability or dependence in activities of daily living. Secondary clinically important outcomes of all-cause mortality and risk of symptomatic intracerebral hemorrhage were also explored.

## Methods

This meta-analysis was performed in accordance with the Preferred Reporting Items for Systematic Reviews and Meta-Analyses (PRISMA) guidelines [18].

### Data Sources and Study Selection

Two investigators independently and in duplicate searched MEDLINE, Embase, Cochrane Central Register of Controlled Trials, Web of Science, and National Institutes of Health ClinicalTrials.gov for studies published on or before 27 November 2015. Queried Medical Subject Headings or keywords included: “ischemic stroke”, “brain infarct”, “endovascular therapy”, “intra-arterial”, “intra-venous”, “fibrinolysis”, and “thrombolysis”. No language restrictions were applied. Searches were limited to randomized controlled trials in human subjects 18 years or older. Exact search terms with search structure and limits are provided in S1 Text. A manual search of references from all articles meeting eligibility, along with relevant review articles, systematic reviews and meta-analyses, was also conducted.

Studies were eligible for inclusion if they were prospective randomized controlled trials evaluating endovascular therapy using stent-retrievers compared to medical management, which we defined as IV t-PA unless contraindicated, for at least one primary or secondary outcome in acute ischemic stroke patients. For trials that produced multiple publications, data from the most recent or the most complete publication were included in the analysis. Studies were excluded if odds ratios and variances (or information to calculate these measures) were not reported for any of the study outcomes; if endovascular therapy (defined as predominant use of second-generation mechanical thrombectomy devices) was not part of the intervention; if treatment allocation was not random; if participants were younger than 18 years; or if follow-up was not at least 90 days.

Two investigators independently and in duplicate screened titles and abstracts of potentially relevant references that were identified by the literature search to evaluate topical relevance and potential eligibility. Articles that passed initial screening were retrieved in full-text and reviewed by two investigators independently and in duplicate to determine eligibility and inclusion for systematic review and meta-analysis. Discrepancies were resolved through discussion and consensus with other investigators.

An *a priori* protocol was developed and provided online at http://www.chadkbush.com/ETAIS/protocol.pdf and is also provided in supporting information as S2 Text.

### Data Abstraction

Investigators independently abstracted all data using a standardized data collection form. The results of data abstraction were compared and discrepancies were resolved through discussion and consensus with other investigators. Trial characteristics abstracted included design of the randomized controlled trial, randomization method and adequacy, blinding method and adequacy, type of control treatment (IV t-PA only or IV t-PA if candidates), number of treatment groups, description of treatment regimens, description of inclusion and exclusion criteria, and demographic characteristics of study populations at baseline, average time from stroke onset to groin puncture, average time from stroke onset to reperfusion, study duration and duration of follow-up, response rate (e.g., withdrawals and dropouts), and adherence to intention-to-treat principle.

The primary pre-specified outcome measure collected was modified Rankin Scale (mRS) scores at 90 days. The mRS is a 7-point scale ranging from 0 (no symptoms) to 6 (death). A score of 2 or less indicates functional independence [19]. For mRS scores, adjusted common odds ratios, derived from ordinal logistic regression models indicating a favorable shift in mRS score distribution (shift analysis), along with standard errors, confidence intervals, and/or p-values were collected, if reported. If shift analysis was not performed, numbers or proportions of participants reported for each of the seven mRS scale categories at 90 days of follow-up for each arm were collected, and unadjusted common odds ratios were calculated from these data. For the primary outcome, adjusted odds ratios and risk ratios for functional independence, indicated as a modified Rankin Scale score of less than or equal to 2, at 90 days, along with standard errors, confidence intervals, and /or p-values, were also collected. If a study did not provide an adjusted effect estimate, unadjusted effect measures were calculated from the abstracted data.

The pre-specified secondary outcomes included all-cause mortality and risks of symptomatic intra-cerebral hemorrhage for treatment arms. For all other outcomes, number of events and participants in each arm and reported measures of association, along with standard errors, confidence intervals, and/or p-values, were collected. For studies that reported different sub-types of symptomatic intra-cerebral hemorrhage, the total events of all types were collected.

### Quality Assessment

Trial quality was assessed using a domain-based approach recommended by the Cochrane Collaboration [20]. This evaluation method includes assessment of the randomization process, allocation concealment, blinding procedures, withdrawals and dropouts, and the conduct of intention-to-treat analysis.

### Statistical Analysis

The primary effect measure of interest was the pooled common [13, 14, 17, 21] odds ratio for a shift in the direction of better functional outcome on the modified Rankin Scale at 90 days. If the common odds ratio from ordinal mRS shift analysis was not provided [15, 16], it was estimated by fitting an unadjusted ordinal logistic regression model using the number of participants reported for each of the seven mRS scale categories at 90 days for each arm, with calculation of a 95% confidence interval to indicate statistical precision.

If the adjusted odds ratio and risk ratio for functional independence were not provided, unadjusted effect estimates were calculated, along with 95% confidence intervals, using the number of participants reported for mRS score categories 0, 1, and 2 at 90 days and total number of participants for each arm. The secondary effect measures of interest included odds ratios for all-cause mortality and symptomatic intracerebral hemorrhage. If these measures were not directly provided by the trials included, they were calculated along with their 95% confidence intervals using the number of events and participants in each trial arm.

Prior to pooling, common odds ratio, odds ratio, and risk ratio estimates were logarithmically transformed to normalize their distribution and stabilize their variance. Pooled effect estimates were calculated using an inverse-variance weighted restricted maximum-likelihood (REML) random-effects model. REML was chosen *a priori* since the method is highly robust to violations of assumptions that cause other statistical pooling procedures to yield unstable values [22], and heterogeneity was expected due to differences in trial characteristics. Dersimonian and Laird’s Q test was used to assess the presence of heterogeneity of effects, and the I^2^ index statistic was used to quantify the extent of heterogeneity [23, 24].

Subgroup analyses were used to explore whether participant characteristics or trial features influenced effect estimates. Subgroup analyses included the examination of treatment effects according to gender, baseline age (dichotomized as < 70 years versus ≥ 70 years), National Institutes of Health Stroke Scale (NIHSS; range 0 to 42, with higher scores indicating more severe neurologic deficits; dichotomized as < 17 versus ≥ 17), and Alberta Stroke Program Early Computed Tomography Score [25, 26] (ASPECTS; range, 0 to 10, with lower scores given for evidence of early ischemic change in each defined region on the CT scan; dichotomized as < 8 versus ≥ 8), as well as the administration of IV t-PA as medical management (given versus not given). Subgroups were assigned categorical factor labels and modeled as moderators using mixed-effects restricted maximum likelihood meta-regression, which provided omnibus tests of significance for between-group differences.

Meta-regression analyses were used to explore the influence that workflow efficiencies and time to reperfusion had on treatment effect measures. Mixed-effects restricted maximum likelihood models were constructed using median times from stroke onset to groin puncture and from stroke onset to reperfusion as moderators. Effect estimate response variables were log-transformed common odds ratios for a beneficial mRS shift due to endovascular therapy.

Although recent trials involving acute ischemic stroke patients have begun using mRS shift analysis [13–15, 17, 21], previous trials of first generation mechanical thrombectomy devices commonly reported odds ratios or risk ratios for the dichotomous outcome of functional independence, defined as mRS ≤ 2 (as opposed to functional dependence, mRS ≥ 3). To assess for consistency with our primary outcome of a shift in the ordinally treated mRS score distributions and to allow comparison of our findings with those of previous trials and meta-analyses, we also examined the dichotomous functional independence outcome as a sensitivity analysis.

Influence analyses for primary and secondary outcomes were also performed to assess the impact of individual studies on the overall pooled estimates. Publication bias was assessed with visual inspection of funnel plots on which standard errors were plotted against effect sizes. Duval and Tweedie’s nonparametric trim-and-fill method was employed to detect and estimate effects of missing studies [27]. Both Kendall’s rank correlation statistic and Egger’s mixed regression tests for funnel plot asymmetry were calculated to assess for statistically significant funnel plot asymmetry [28]. All statistical analyses were conducted in R 3.1.3 software, with restricted maximum likelihood random effects models fit using the Metafor package [29].

## Results

### Study Selection

**Fig 1:**
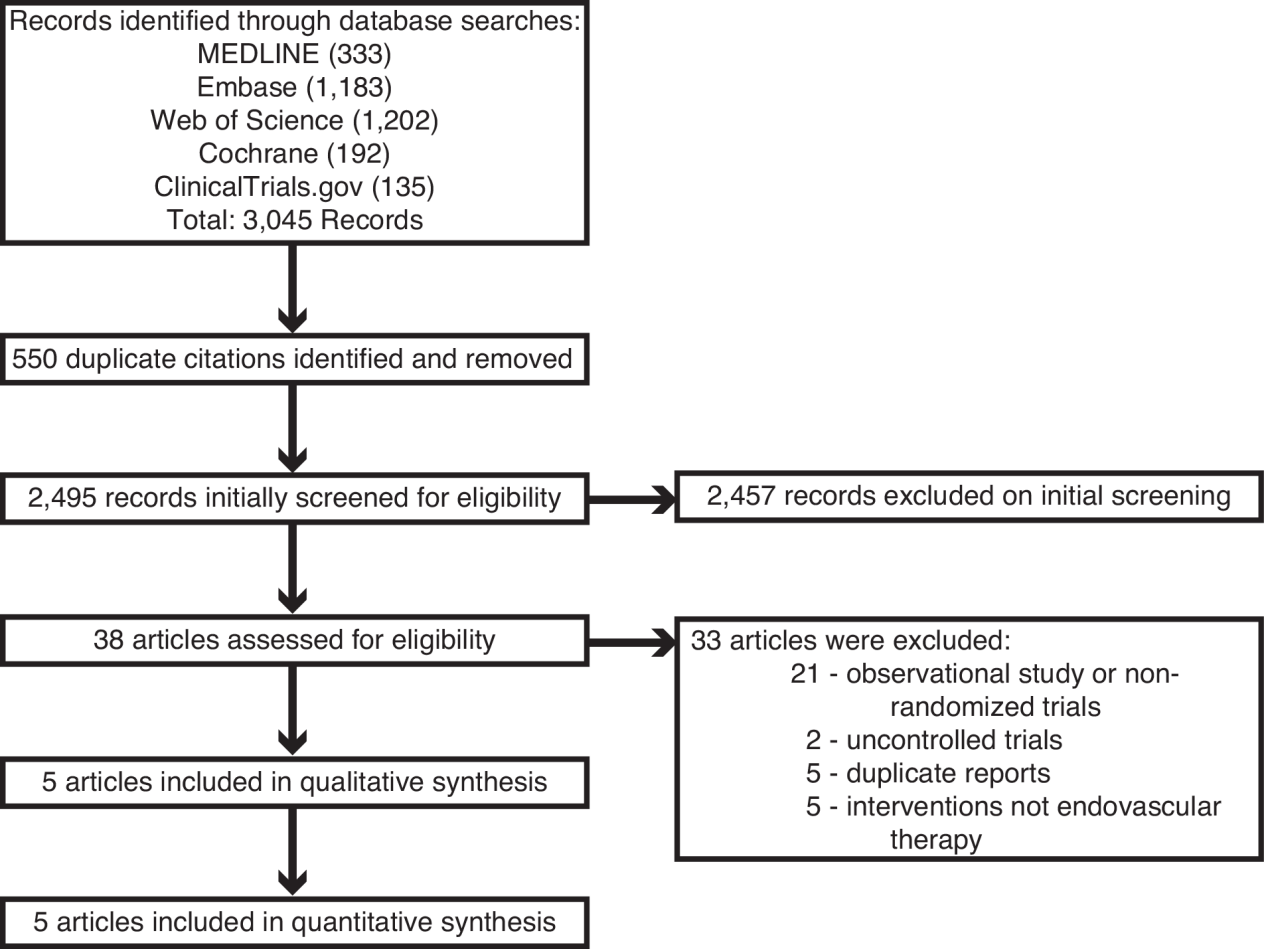
Flow selection of randomized controlled trials included in the meta-analysis.

Our initial search strategy retrieved a total of 3,045 citations. After removing 550 duplicate citations, the titles and abstracts of 2,495 articles were initially screened. Of those, 38 articles were assessed for eligibility, and 33 articles were excluded for not meeting inclusion criteria: 21 presented data obtained from observational studies or nonrandomized trials; 2 presented data from uncontrolled trials; 5 presented duplicate reports of data from previous trials; 5 presented data from randomized controlled trials whose intervention did not include endovascular therapy with predominant use of second-generation mechanical thrombectomy devices. 5 reports [13–17] of randomized controlled trials were included in both the qualitative synthesis and meta-analysis (Fig 1).

### Study Characteristics

Tables 1 and 2 describe characteristics of trials and included patients, respectively, for the randomized controlled trials included in the meta-analysis. The five included trials presented data from 1,287 patients. 653 (50.7%) were randomized to control arms with medical management alone, and 573 (87.7%) medical management patients received IV t-PA. 634 (49.3%) patients were randomized to endovascular therapy. 526 (83.0%) patients received IV t-PA in addition to endovascular intervention and 536 (84.5%) received endovascular intervention with a stent-retriever. Importantly, 373 (80.4%) of patients receiving endovascular treatment were documented as having achieved good reperfusion, defined as modified Thrombolysis in Cerebral Infarction (mTICI) score of 2b or 3. Median onset-to-groin-puncture times ranged from 200 to 269 minutes, and median onset-to-reperfusion times ranged from 241 to 355 minutes.

**Table 1:** Characteristics of Randomized Trials Included in the Meta-Analysis.

		Medical Management Arm			Endovascular Arm						
Trial, Publication Year	No. of Patients	Primary Treatment Modalities	Patients, N (%)	IV t-PA, N (%)	Primary Treatment Modalities	Patients, N (%)	IV t-PA, N (%)	Stent-Retriever Deployed, N (%)	mTICI 2b or 3 (Good Reperfusion), N (%)	Onset-to-Groin-Puncture Time, min (IQR)	Onset-to-Reperfusion Time, min (IQR)
MR CLEAN, 2014 [13]	500	IV t-PA if candidates^a^	267 (53.4%)	242 (90.6%)	IA thrombolysis^b^ and/or mechanical thrombectomy^c^ + IV t-PA if candidates^a^	233 (46.6%)	203 (87.1%)	190 (81.5%)	115 (58.7%)	260 (210–313)	332 (279–394) [30]
ESCAPE, 2015 [14]	315	IV t-PA if candidates^a^	150 (47.6%)	118 (78.7%)	Mechanical thrombectomy + IV t-PA if candidates^a^	165 (52.4%)	120 (72.7%)	130 (78.8%)	113 (72.4%)^d^^,^ ^e^	200 (116–315) [31]	241(176–359)^f^
EXTEND-IA, 2015 [15]	70	IV t-PA alone	35 (50%)	35 (100%)	Mechanical thrombectomy + IV t-PA	35 (50%)	35 (100%)	31 (88.6%)	25 (86.2%)^d^	210 (83–159)	248 (204–277)^g^
SWIFT PRIME, 2015 [16]	196	IV t-PA alone	98 (50%)	98 (100%)	Mechanical thrombectomy + IV t-PA	98 (50%)	98 (100%)	87 (88.8%)	53 (83.0%)	224 (165–275)	252 (190–300)^h^
REVASCAT, 2015 [17]	206	IV t-PA if candidates^a^	103 (50%)	80 (77.7%)	Mechanical thrombectomy + IV t-PA if candidates^a^	103 (50%)	70 (68%)	98 (95.1%)	67 (65.7%)	269 (201–340)	355 (269–430)^e^
OVERALL	1,287		653 (50.7%)	573 (87.7%)		634 (49.3%)	526 (83.0%)	536 (84.5%)	373 (80.4%)	200 to 269 min	241 to 355 min

^a^ If participants were not a candidate for IV t-PA, they were given antithrombotic and supportive therapies.

^b^ Use of either alteplase or urokinase for intraarterial thrombolysis was allowed in this trial.

^c^ Mechanical treatment could involve thrombus retraction, aspiration, wire disruption or use of a retrievable stent.

^d^ Percentage of patients who achieved a final score on mTICI of 2b or 3 (good reperfusion) of those randomized to endovascular therapy who had an initial occlusion on angiography.

^e^ For ESCAPE, the threshold for 2b reperfusion was set higher at >66%, compared to >50% in the other trials.

^f^ Defined as time from stroke onset to first reperfusion.

^g^ Defined as time from stroke onset to achievement of good reperfusion, as defined by an mTICI score of 2b or 3.

^h^ Described as time from stroke onset to first deployment of stent-retriever. Abbreviations: IV, intravenous; t-PA, tissue plasminogen activator; mTICI, modified Thrombolysis in Cerebral Infarction score [32]; IQR, inter-quartile range; MR CLEAN, Multicenter Randomized Clinical Trial of Endovascular Treatment for Acute Ischemic Stroke in the Netherlands [13]; ESCAPE, Endovascular Treatment for Small Core and Anterior Circulation Proximal Occlusion with Emphasis on Minimizing CT to Recanalization Times [14]; EXTEND-IA, Extending the Time for Thrombolysis in Emergency Neurological Deficits–Intra-Arterial [15]; SWIFT PRIME, Solitaire with the Intention for Thrombectomy as Primary Endovascular Treatment [16]; REVASCAT, Randomized Trial of Revascularization with Solitaire FR Device versus Best Medical Therapy in the Treatment of Acute Stroke Due to Anterior Circulation Large Vessel Occlusion Presenting within Eight Hours of Symptom Onset [17].

**Table 2:** Characteristics of included patients for randomized controlled trials.

		Medical Management Arm					Endovascular Arm				
Trial	No. of Patients	Patients, N (%)	Female, N (%)	Age, y (IQR or ±SD)	NIHSS Score (IQR)	ASPECTS (IQR)	Patients, N (%)	Female, N (%)	Age, y (IQR or ±SD)	NIHSS Score (IQR)	ASPECTS (IQR)
MR CLEAN, 2014 [13]	500	267 (53.4%)	110 (41.2%)	65.7 (55.5–76.4)	18 (14–22)	9 (8–10)	233 (46.6%)	98 (42.1%)	65.8 (54.5–76)	17 (14–21)	9 (7–10)
ESCAPE, 2015 [14]	315	150 (47.6%)	79 (52.7%)	70 (60–81)	17 (12–22)	9 (8–10)	165 (52.4%)	86 (52.1%)	71 (60–81)	16 (13–20)	9 (8–10)
EXTEND-IA, 2015 [15]	70	35 (50%)	18 (51.4%)	70.2 (± 11.8)	13 (9–19)	Not Reported	35 (50%)	18 (51.4%)	68.6 (±12.3)	17 (13–20)	Not Reported
SWIFT PRIME, 2015 [16]	196	98 (50%)	51 (52%)	66.3 (± 11.3)	17 (13–19)	9 (8–10)	98 (50%)	44 (44.9%)	65 (± 12.5)	17 (13–20)	9 (7–10)
REVASCAT, 2015 [17]	206	103 (50%)	49 (47.6)	67.2 (± 9.5)	17 (12–19)	8 (6–9)	103 (50%)	48 (46.6%)	65.7 (± 11.3)	17 (14–20)	7 (6–9)
OVERALL	1,287	653 (50.7%)	307 (47.0%)	65.7 to 70.2 years	13 to 18	8 to 9	634 (49.3%)	294 (46.4%)	65 to 71 years	16 to 17	7 to 9

Abbreviations: IQR, inter-quartile range; SD, standard deviation; NIHSS, National Institutes of Health Stroke Scale; ASPECTS, Alberta Stroke Program Early Computed Tomography Score [25, 26]; MR CLEAN, Multicenter Randomized Clinical Trial of Endovascular Treatment for Acute Ischemic Stroke in the Netherlands [13]; ESCAPE, Endovascular Treatment for Small Core and Anterior Circulation Proximal Occlusion with Emphasis on Minimizing CT to Recanalization Times [14]; EXTEND-IA, Extending the Time for Thrombolysis in Emergency Neurological Deficits–Intra-Arterial [15]; SWIFT PRIME, Solitaire with the Intention for Thrombectomy as Primary Endovascular Treatment [16]; REVASCAT, Randomized Trial of Revascularization with Solitaire FR Device versus Best Medical Therapy in the Treatment of Acute Stroke Due to Anterior Circulation Large Vessel Occlusion Presenting within Eight Hours of Symptom Onset [17].

307 (47.0%) participants randomized to medical management and 294 (46.4%) participants randomized to endovascular therapy were female. The number of participants in each trial ranged from 70 to 500. Trial quality was generally high (Table 3), with all trials employing appropriate randomization procedures, allocation concealment, blinding of outcome assessment, and intention-to-treat analysis. Due to the nature of the intervention, none of the trials employed blinding of study participants.

**Table 3:** Judgment results from domain-based assessments of risks of bias for included studies.

	Sequence Generation	Allocation Concealment	Blinding of Participants and Personnel	Blinding of Outcome Assessment	Incomplete Outcome Data	Selective Outcome Reporting	Other Issues
Trial, Publication Year	Selection Bias	Selection Bias	Performance Bias	Detection Bias	Attrition Bias	Reporting Bias	
MR CLEAN, 2014 [13]	Low risk.	Low risk.	High risk.	Low risk.	Low risk.	Low risk.	Low risk.
ESCAPE, 2015 [14]	Low risk.	Low risk.	High risk.	Low risk.	Low risk.	Low risk.	Low risk.
EXTEND-IA, 2015 [15]	Low risk.	Low risk.	High risk.	Low risk.	Low risk.	Low risk.	Low risk.
SWIFT PRIME, 2015 [16]	Low risk.	Low risk.	High risk.	Low risk.	Low risk.	Low risk.	Low risk.
REVASCAT, 2015 [17]	Low risk.	Low risk.	High risk.	Low risk.	Low risk.	Low risk.	Low risk.

Abbreviations: NIHSS, National Institutes of Health Stroke Scale; ASPECTS, Alberta Stroke Program Early Computed Tomography Score [25, 26]; MR CLEAN, Multicenter Randomized Clinical Trial of Endovascular Treatment for Acute Ischemic Stroke in the Netherlands [13]; ESCAPE, Endovascular Treatment for Small Core and Anterior Circulation Proximal Occlusion with Emphasis on Minimizing CT to Recanalization Times [14]; EXTEND-IA, Extending the Time for Thrombolysis in Emergency Neurological Deficits–Intra-Arterial [15]; SWIFT PRIME, Solitaire with the Intention for Thrombectomy as Primary Endovascular Treatment [16]; REVASCAT, Randomized Trial of Revascularization with Solitaire FR Device versus Best Medical Therapy in the Treatment of Acute Stroke Due to Anterior Circulation Large Vessel Occlusion Presenting within Eight Hours of Symptom Onset [17].

### Primary and Secondary Outcomes

**Fig 2:**
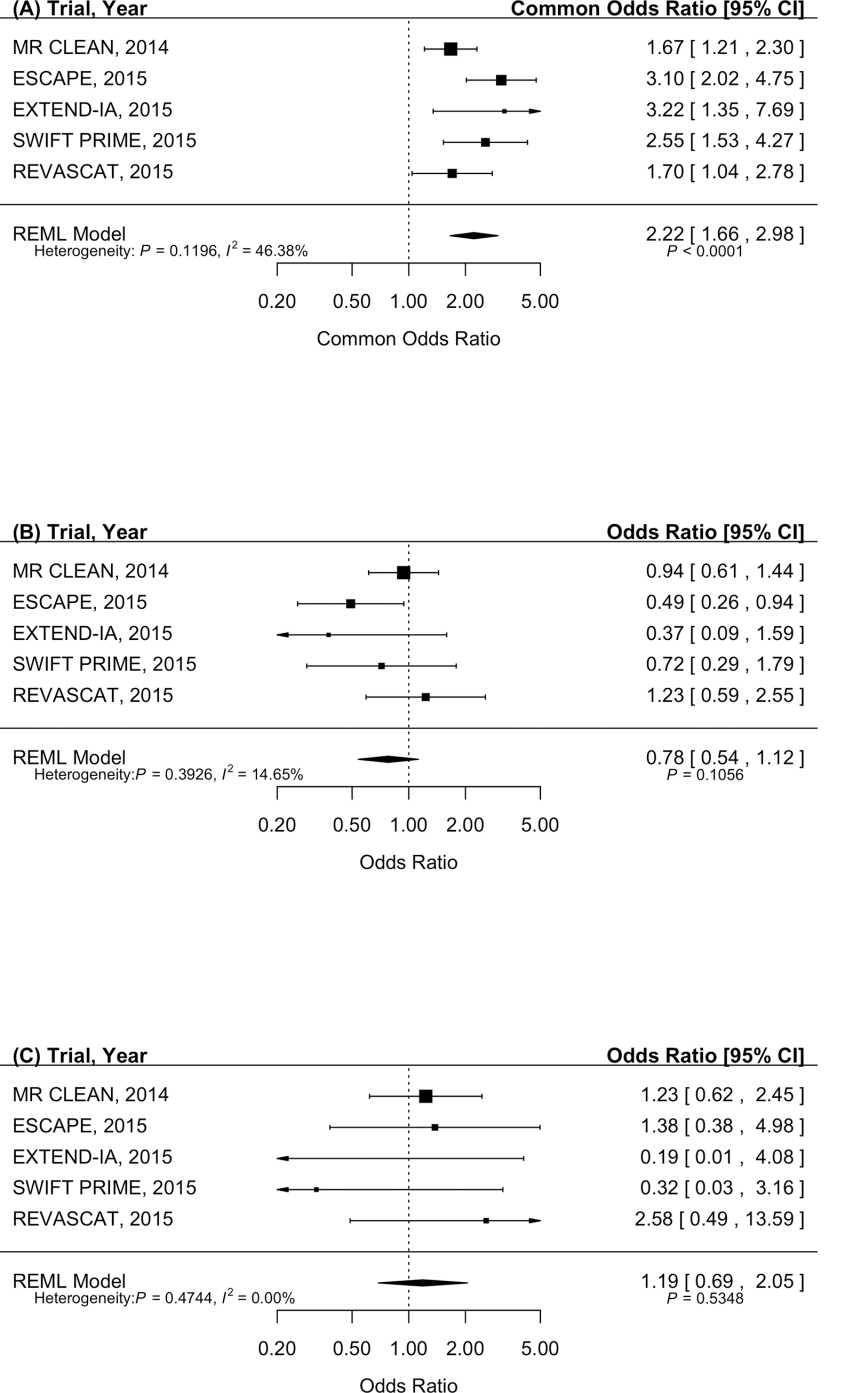
Pooled effect estimates by restricted maximum likelihood random effects model with inverse variance weighting. (A) Primary outcome of a shift in scores on modified Rankin Scale at 90 days between endovascular and medical management (common odds ratio, indicating odds of a more favorable distribution of scores on the modified Rankin Scale). (B) Secondary outcome of all-cause mortality (odds ratios). (C) Secondary outcome of symptomatic intra-cerebral hemorrhage (odds ratios).

All five included studies presented information for the primary outcome of mRS score at 90-days of follow-up and for the secondary outcomes of all-cause mortality and symptomatic intracerebral hemorrhage (Fig 2A–2C). Overall, patients randomized to endovascular therapy had 2.22 times greater odds of a more favorable mRS score at 90-days post-stroke compared to medical management (95% CI, 1.66 to 2.98; P < 0.0001). Although the p-value for the Dersimonian and Laird Q test was not significant (P = 0.1196), the *I*^*2*^ statistic suggested the presence of modest statistical heterogeneity between the trials (*I*^*2*^ = 46.38%).

For mortality at 90 days, no significant effect of endovascular therapy compared to medical management was identified. The pooled odds ratio for mortality was 0.78 (95% CI, 0.54 to 1.12; P = 0.1056) with no statistically significant heterogeneity between trials. No statistically significant effect on symptomatic intracerebral hemorrhage was found following endovascular therapy compared to medical management. The pooled odds ratio for symptomatic intracerebral hemorrhage was 1.19 (95% CI, 0.69 to 2.05; P = 0.5348) with no statistically significant heterogeneity between trials.

### Subgroup, Meta-Regression and Sensitivity Analyses

Subgroup analyses are presented in Table 4. Endovascular therapy was associated with better functional outcome across all subgroups without evidence of heterogeneity of effect. There were no differences in the associations of endovascular therapy with better functional outcome by patient gender [OR (95% CI) for males and females: 2.60 (1.65, 4.10) and 2.53 (1.63, 3.90), respectively; P for subgroup differences = 0.9255] or by patient age [OR (95% CI) for < 70 years and ≥ 70 years: 2.41 (1.51, 3.84) and 2.26 (1.20, 4.26), respectively; P for subgroup differences = 0.8783]. Endovascular therapy was also equally effective for moderate and severe strokes [OR (95% CI) for NIHSS < 17 and ≥ 17: 1.77 (1.22, 2.58) and 2.23 (1.58, 3.15), respectively; P for subgroup differences = 0.3761] and regardless of the presence of early ischemic changes on CT as measured by ASPECTs score dichotomized at 8 [OR (95% CI) for ASPECTS < 8 and ≥ 8: 1.82 (1.19, 2.79) and 2.19 (1.61, 2.98); P for subgroup differences = 0.5274]. Endovascular intervention retained its positive association with better functional outcome for patients who did not receive IV t-PA [OR (95% CI): 2.41 (1.76, 3.31)] as well as for patients who did receive IV t-PA [OR (95% CI): 1.85 (1.39, 2.46)]. These effects were not statistically heterogeneous (P for subgroup differences = 0.1884].

**Table 4:** Subgroup analyses.

	Ordinal Analysis of mRS Scores at 90 Days			
	Number of Studies	Pooled OR [95% CI]	Effect P	Subgroup P^a^
Overall Analysis	5	2.22 [1.66, 2.98]	<0.0001	
Gender				
Male	2 [14, 16]	2.60 [1.65, 4.10]	<0.0001	0.9255
Female	2 [14, 16]	2.53 [1.63, 3.90]	<0.0001	
Age				
< 70 years	2 [16–17]	2.41 [1.51, 3.84]	0.0002	0.8783
≥ 70 years	4 [13–14, 16–17]	2.26 [1.20, 4.26]	0.0113	
NIHSS Score				
< 17	3 [13, 16–17]	1.77 [1.22, 2.58]	0.0028	0.3761
≥ 17	4 [13–14, 16–17]	2.23 [1.58, 3.15]	<0.0001	
ASPECTS Score				
Low (< 8)	4 [13–14, 16–17]	1.82 [1.19, 2.79]	0.0061	0.5274
High (≥ 8)	4 [13–14, 16–17]	2.19 [1.61, 2.98]	<0.0001	
IV Alteplase				
Given	3 [13–14, 17]	1.85 [1.39, 2.46]	<0.0001	0.1884
Not Given	5 [13–17]	2.41 [1.76, 3.31]	<0.0001	

^a^ P-values for subgroup differences, i.e. omnibus test of moderator coefficients from mixed-effects meta-regression models. Abbreviations: mRS, modified Rankin Scale; NIHSS, National Institutes of Health Stroke Scale; ASPECTS, Alberta Stroke Program Early Computed Tomography Score [25, 26]; IV, intravenous.

**Fig 3:**
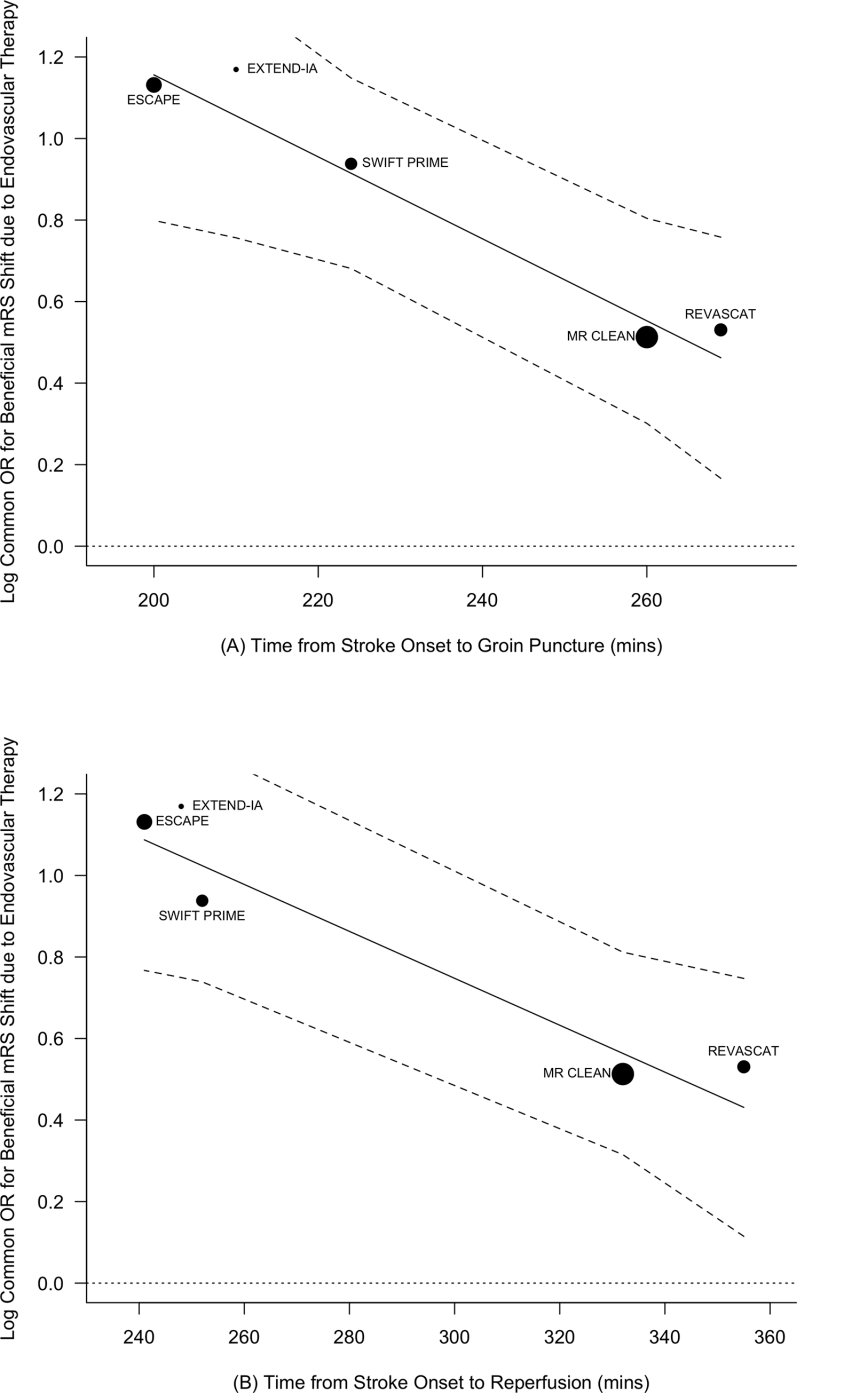
Meta-regression analysis relating trial effect estimates to workflow efficiencies. Mixed effects restricted maximum likelihood meta-regression models of log common odds ratios for improved functional outcome regressed against (A) median time from stroke onset to groin puncture and (B) median time from stroke onset to reperfusion, indicating that improved workflow efficiencies significantly influence the beneficial effects of endovascular treatment (P = 0.0077 and 0.0089, respectively).

Inverse-variance weighted meta-regressions of our observed log odds ratios for improvement in mRS score against workflow efficiency measures are shown in Fig 3. Both median study times from stroke onset to groin puncture and from stroke onset to reperfusion showed significant (P = 0.0077 and P = 0.0089, respectively) inverse associations with better functional outcomes (Fig 3A and 3B, respectively). Shorter times from stroke onset to groin puncture and to reperfusion result in higher efficacy of endovascular therapy on functional outcomes.

**Fig 4:**
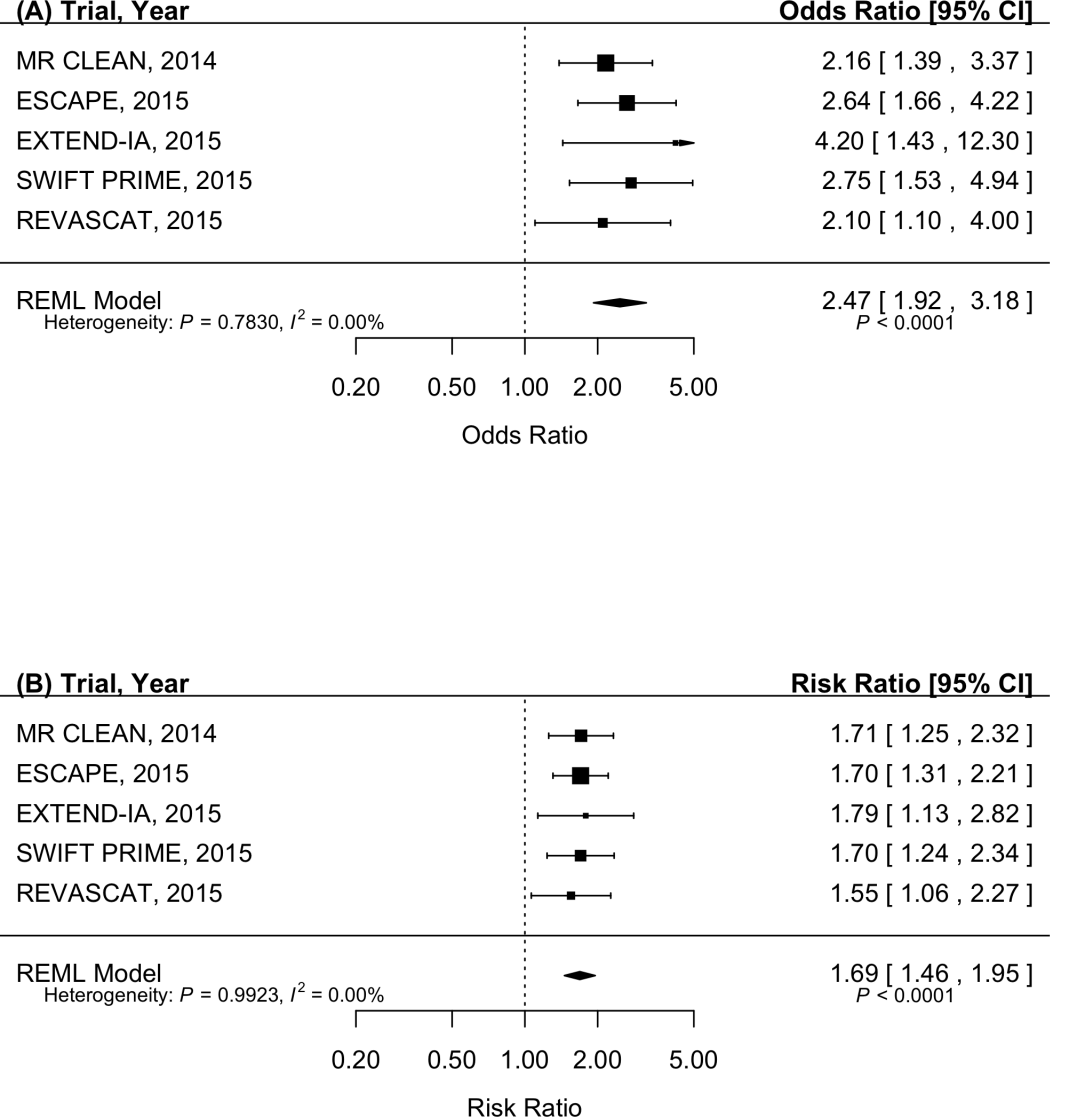
Forest plots of meta-analyses for pooled odds ratios and risk ratios for functional independence (modified Rankin Scale scores of 0 to 2). Patients randomized to endovascular intervention with retrievable stents have (A) 2.47 (95% CI: 1.92 to 3.18) times greater odds and (B) 1.69 (95% CI: 1.46 to 1.95) times greater probability of experiencing functional independence at 90-days post-stroke compared to those randomized to medical management.

Results from our functional independence (mRS ≤ 2) outcome sensitivity analysis (Fig 4A) were similar to those of the mRS shift analysis, with a pooled odds ratio (95% CI) of 2.47 (1.92, 3.18). Given that odds ratios have inherent mathematical bias away from the null compared to risk ratios, the results of the functional independence sensitivity analysis with pooled risk ratios were expectedly conservative compared to analyses pooling odds ratio estimates (Fig 4B). The pooled risk ratio (95% CI) for achieving functional independence at 90-days was 1.69 (1.46, 1.95).

**Fig 5:**
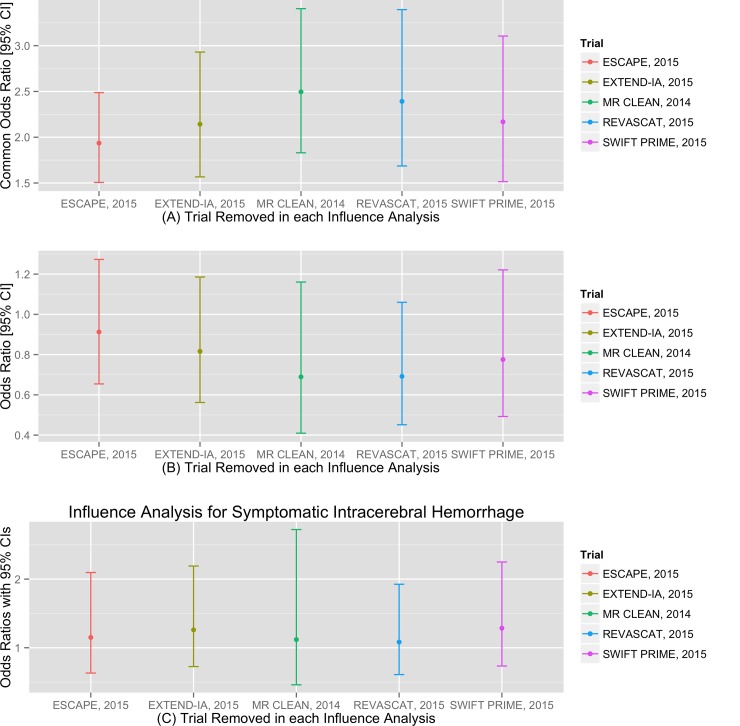
Influence analyses for pooled effects on primary and secondary outcomes. Removal of any single trial does not significantly influence the pooled effect of endovascular therapy on (A) the primary outcome of a beneficial shift in mRS score distributions, (B) the secondary outcome of mortality or (C) the secondary outcome of symptomatic intra-cerebral hemorrhage.

Influence analyses did not identify any single trial that substantively influenced results for the pooled primary or secondary outcomes (Fig 5).

### Publication Bias

Assessment of funnel plots visually and statistically by Duvall and Tweedie’s nonparametric trim-and-fill method for asymmetry in pooled effects of endovascular therapy on the primary outcome of better functional outcome and secondary outcomes of all-cause mortality and symptomatic intra-cerebral hemorrhage risk indicated that there was no evidence of publication bias (S1, S2, S3, S4, S5 and S6 Figs). Furthermore, Kendall’s Rank Correlation Test and Egger’s Mixed-Effects Regression Test for plot asymmetry were not significant for all primary and secondary outcomes.

Datasets containing abstracted data and R scripts for all statistical analyses performed are provided in supplementary materials, S1 Data and Analyses.

## Discussion

This study reports analyses of 5 recently published randomized controlled trials comparing endovascular therapy to medical management for patients with acute ischemic stroke. The results of this meta-analysis demonstrate improved functional outcomes and higher rates of functional independence at 90 days for acute ischemic stroke patients treated with endovascular therapy with predominant use of second-generation mechanical thrombectomy devices compared with medical management alone. Additionally, this study found no significant differences in symptomatic intracerebral hemorrhage or 90 day all-cause mortality between endovascular therapy and medical management of stroke patients. As opposed to to prior trials of endovascular interventions in stroke [8–10], the five included trials provide a high level of evidence strongly supporting the efficacy of endovascular therapy.

Specifically, patients randomized to endovascular therapy in these trials experienced more than a twofold increase in odds of achieving a beneficial shift in mRS score at 90-days post-stroke compared to those randomized to medical management. Treatment effects were similar regardless of patient gender or age, stroke severity, or the presence of early ischemic changes on imaging studies. Importantly, treatment effects did not differ on the basis of IV t-PA administration. In aggregate, these findings have important implications for the clinical management of stroke, suggesting that endovascular therapy, as an adjunct to medical management, is an important strategy for improving functional outcomes among acute ischemic stroke patients who qualify.

The history of endovascular therapy for acute ischemic stroke has been controversial. In 2013, three trials [8–10] of first-generation mechanical thrombectomy devices for acute ischemic stroke were published showing no benefit of endovascular therapy on functional or clinical outcomes. With their publications, IMS III [8], SYNTHESIS Expansion [9], and MR RESCUE [10], caused a noticeable increase in clinical skepticism regarding the utility of endovascular therapy for acute ischemic stroke. This skepticism was enhanced by subsequent meta-analyses [11–12] emphasizing the lack of efficacy of endovascular treatment in acute ischemic stroke patients. These trials had several notable limitations including: the selection of patients without confirmation of intervention-appropriate occlusions (large-vessel anterior-circulation occlusions), use of less effective intervention modalities including administration of intra-arterial thrombolytics alone or use of first-generation mechanical thrombectomy devices alone, less emphasis on work flow efficiencies to speed the deployment of endovascular treatment, and varied use of IV t-PA as an adjunct to endovascular intervention, especially in the SYNTHESIS Expansion[9] trial whose design was truly that of a comparative effectiveness study between endovascular treatment and IV t-PA. In contrast, the five trials included in this meta-analysis all required demonstration of an appropriate large-vessel occlusion on initial imaging for inclusion, emphasized speed and efficiency in deploying endovascular treatment once a patient was confirmed for inclusion, and had consistent use of IV t-PA in both endovascular therapy and medical management arms.

The current analysis demonstrated a beneficial effect of earlier times from stroke onset to groin puncture and from stroke onset to reperfusion on functional outcomes in ischemic stroke patients. Among the included trials, time to groin puncture ranged from 200 to 269 minutes and time to reperfusion ranged from 241 to 355 minutes. The analyses of these data presented in Fig 3 suggest that delays of even 50 minutes in treatment initiation may have meaningful negative consequences on patient functional outcomes. While this is an intuitive finding since earlier intervention and reperfusion salvages ischemic brain tissue, it does highlight the importance of the rapid delivery of effective endovascular therapy. In 2014, only 56% of the US population had access within 60 minutes to a hospital with neurointerventional capabilities [33]. In this setting, our findings emphasize the public health need for increased access to acute care hospitals with primary or comprehensive stroke center designations and neurointerventional capabilities.

Since we first conducted this meta-analysis in May 2015, two other comparable study-level analyses of endovascular therapy have been published in the literature [34, 35]. The primary results of our analysis agree with those published in both these articles. Still, our analysis is novel in that we were able to perform meta-regression analyses to explore the effects of time to treatment and time to reperfusion on functional outcomes after endovascular therapy. Importantly and in agreement with previous summarizations of these data, our subgroup analyses did not detect significant differences in the effects of endovascular therapy according to the type of medical management employed. These findings suggest that endovascular therapy is equally effective as an adjunct to IV t-PA or to medical management using antithrombotics alone when IV t-PA is contraindicated. Another study strength is the inclusion of generally high quality randomized controlled trials, thereby reducing the likelihood that the observed effect of endovascular therapy on stroke outcomes can be explained entirely by bias and confounding. Furthermore, influence analyses supported the robustness of study findings and no publication bias was evident.

Limitations of this meta-analysis should be addressed. While study quality was generally high, participants could not be blinded to intervention in any of the trials, which could have led to performance bias. While we did not find any evidence of heterogeneity of effect across subgroups of gender, age dichotomized at 70, stroke severity by NIHSS dichotomized at 17, or early ischemic evidence on CT by ASPECTS dichotomized at 8, some of these findings could be due in part to trials’ patient inclusion and exclusion criteria. Many of the trials excluded patients with low ASPECTS (< 6), older patients (> 80 years), or patients with pre-stroke mRS ≥ 3, which limit our ability to detect real differences in endovascular treatment benefits for each of these groups. While our study was able to explore the effect of time to treatment and time to reperfusion on effect estimates, we are unable to estimate a precise time after stroke when endovascular therapy becomes futile. Further studies delineating patient subgroups who would most benefit from endovascular treatment and a precise estimate of how much time can lapse after stroke onset before endovascular treatment becomes futile need to be performed.

## Conclusions

This meta-analysis of five prospective randomized controlled trials comparing endovascular therapy using predominantly second-generation mechanical thrombectomy devices as an adjunct to medical management versus medical management alone in acute ischemic stroke demonstrates superior functional outcomes in subjects receiving endovascular therapy. Furthermore, it demonstrates non-inferiority to medical management of acute ischemic stroke in terms of important clinical end points of mortality and symptomatic intracerebral hemorrhage. This meta-analysis supports recommendations for including earlier endovascular therapy as a strategy to improve clinical outcomes in acute ischemic stroke patients with imaging-demonstrated large-vessel anterior-circulation occlusions.

## Supporting Information

S1 Data and Analyses Datasets and R scripts. Datasets containing all abstracted data used in statistical analyses as well as R scripts to perform analyses presented are supplied in this package.
(ZIP)

S1 Fig Funnel plot for assessing publication bias in primary outcome of beneficial shift in mRS score distributions due to endovascular therapy. Both Kendall’s rank correlation and Egger’s mixed regression tests for missing studies were statistically nonsignificant. The single hollow point represents a non-significant result imputed with Duval and Tweedie’s trim and fill method.
(TIF)

S2 Fig Forest plot of primary outcome of beneficial shift in mRS score distributions due to endovascular therapy including trim-and-fill imputed study. Duval and Tweedie’s trim and fill method for imputing missing study results provided a single, non-significant positive result, which has been pooled here with the other included trial results.
(TIF)

S3 Fig Funnel plot for assessing publication bias in secondary outcome of mortality benefit due to endovascular therapy. Both Kendall’s rank correlation and Egger’s mixed regression tests for missing studies were statistically nonsignificant. The single hollow point represents a non-significant result imputed with Duval and Tweedie’s trim and fill method.
(TIF)

S4 Fig Forest plot of secondary outcome of mortality benefit due to endovascular therapy including trim-and-fill imputed study. Duval and Tweedie’s trim and fill method for imputing missing study results provided a single, non-significant positive result, which has been pooled here with the other included trial results.
(TIF)

S5 Fig Funnel plot for assessing publication bias in secondary outcome of symptomatic intracerebral hemorrhage benefit due to endovascular therapy. Both Kendall’s rank correlation and Egger’s mixed regression tests for missing studies were statistically nonsignificant. The single hollow point represents a non-significant result imputed with Duval and Tweedie’s trim and fill method.
(TIF)

S6 Fig Forest plot of secondary outcome of symptomatic intracerebral hemorrhage benefit due to endovascular therapy including trim-and-fill imputed study. Duval and Tweedie’s trim and fill method for imputing missing study results provided a single, non-significant positive result, which has been pooled here with the other included trial results.
(TIF)

S1 Text Search strategy. Exact search terms and syntax used to produce the initial search results.
(DOCX)

S2 Text Project proposal and protocol. The project proposal and *a priori* protocol is provided.
(DOCX)
