# Supplementary material for: Endovascular Treatment with Stent-Retriever Devices for Acute Ischemic Stroke: A Meta-Analysis of Randomized Controlled Trials
Source: PLoS One. 2016 Jan 25;11(1):e0147287. doi: 10.1371/journal.pone.0147287 (PMC4726653; doi:10.1371/journal.pone.0147287)
Supplement: S1 Data and Analyses — Datasets containing all abstracted data used in statistical analyses as well as R scripts to perform analyses presented are supplied in this package. (ZIP) [file pone.0147287.s001.zip › Data and Analyses/Subgroups/04.aspectslow.pdf]

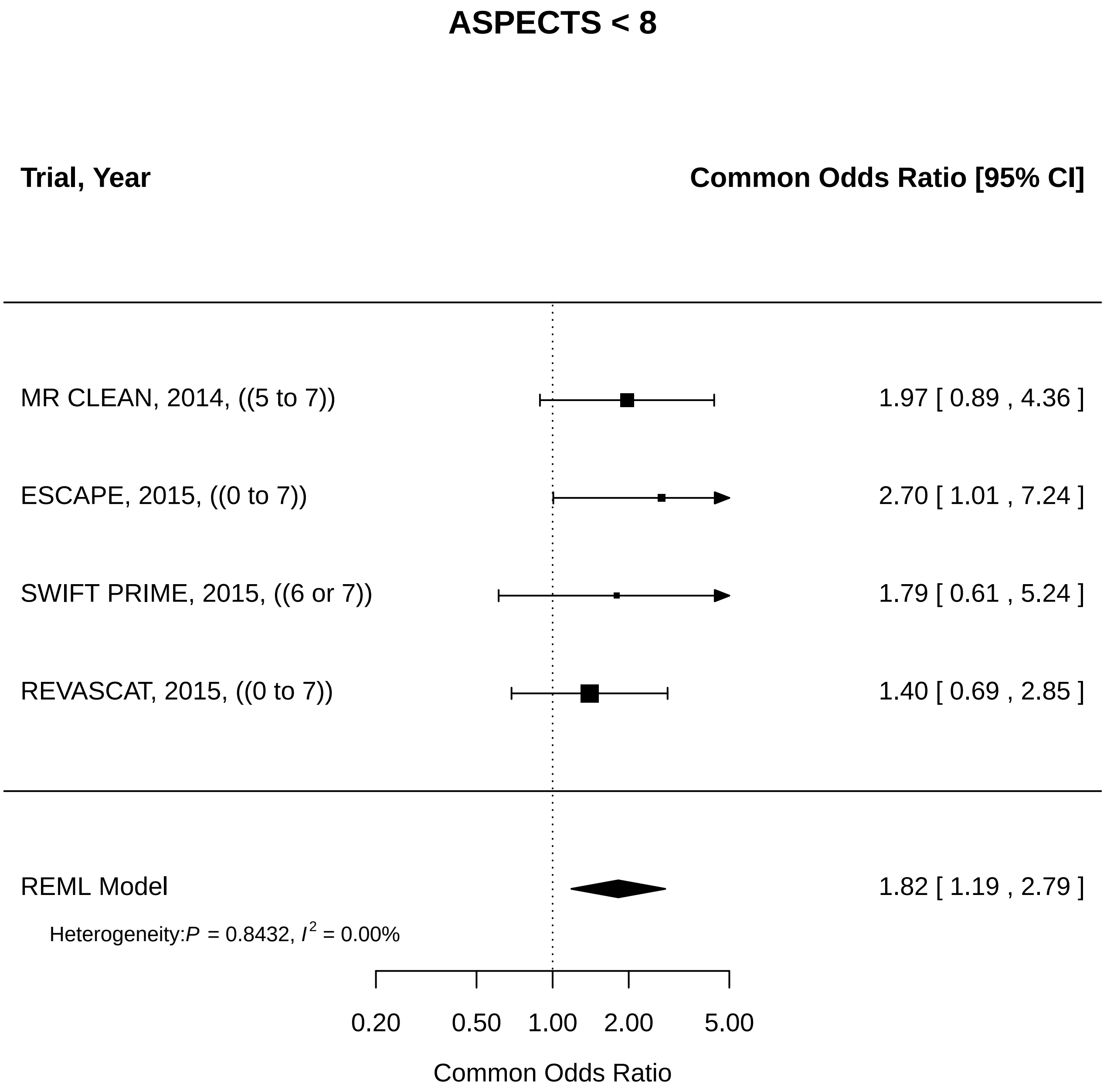
ASPECTS < 8
Trial, Year
Common Odds Ratio [95% CI]
MR CLEAN, 2014, ((5 to 7))
1.97 [0.89 , 4.36]
ESCAPE, 2015, ((0 to 7))
2.70 [1.01 , 7.24]
SWIFT PRIME, 2015, ((6 or 7))
1.79 [0.61 , 5.24]
REVASCAT, 2015, ((0 to 7))
1.40 [0.69 , 2.85]
REML Model
1.82 [1.19 , 2.79]
Heterogeneity: P = 0.8432, I2 = 0.00%
0.20
0.50
1.00
2.00
5.00
Common Odds Ratio
