# Supplementary figures and images for: Endovascular Treatment with Stent-Retriever Devices for Acute Ischemic Stroke: A Meta-Analysis of Randomized Controlled Trials
Source: PLoS One. 2016 Jan 25;11(1):e0147287. doi: 10.1371/journal.pone.0147287 (PMC4726653; doi:10.1371/journal.pone.0147287)

# Females

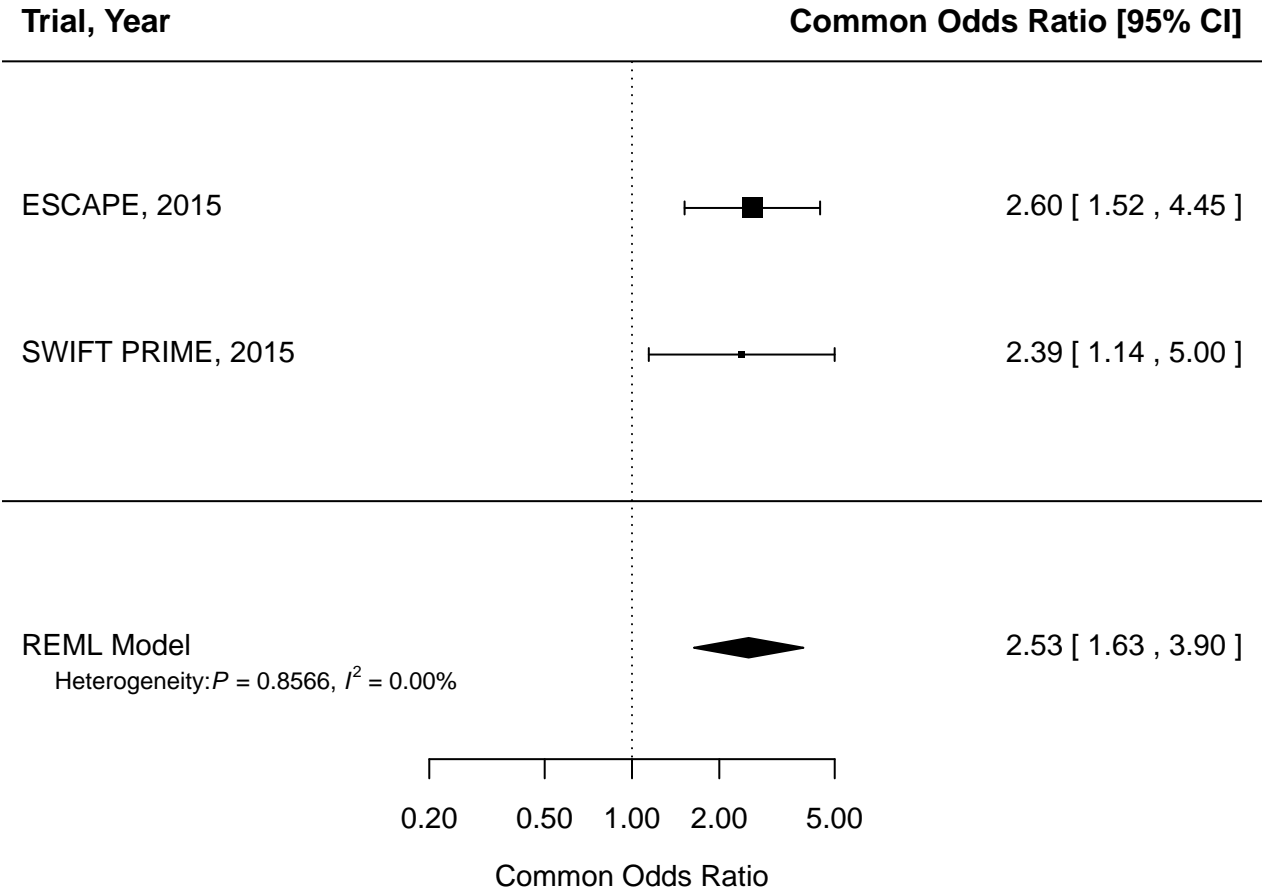

Supplement: S1 Data and Analyses — Datasets containing all abstracted data used in statistical analyses as well as R scripts to perform analyses presented are supplied in this package. (ZIP) [file pone.0147287.s001.zip › Data and Analyses/Subgroups/01.females.pdf]

# Males

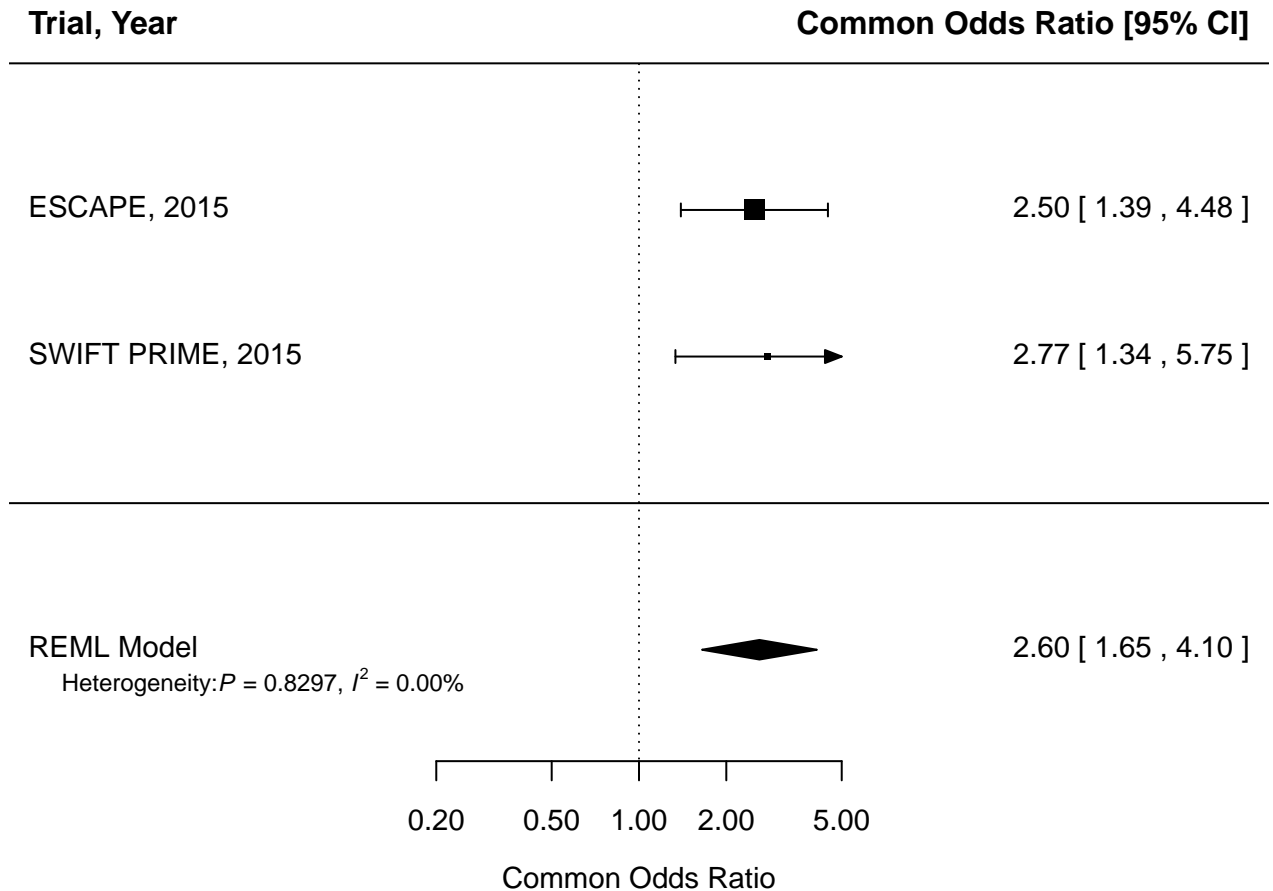

Supplement: S1 Data and Analyses — Datasets containing all abstracted data used in statistical analyses as well as R scripts to perform analyses presented are supplied in this package. (ZIP) [file pone.0147287.s001.zip › Data and Analyses/Subgroups/01.males.pdf]

# Age greater than or equal to 70

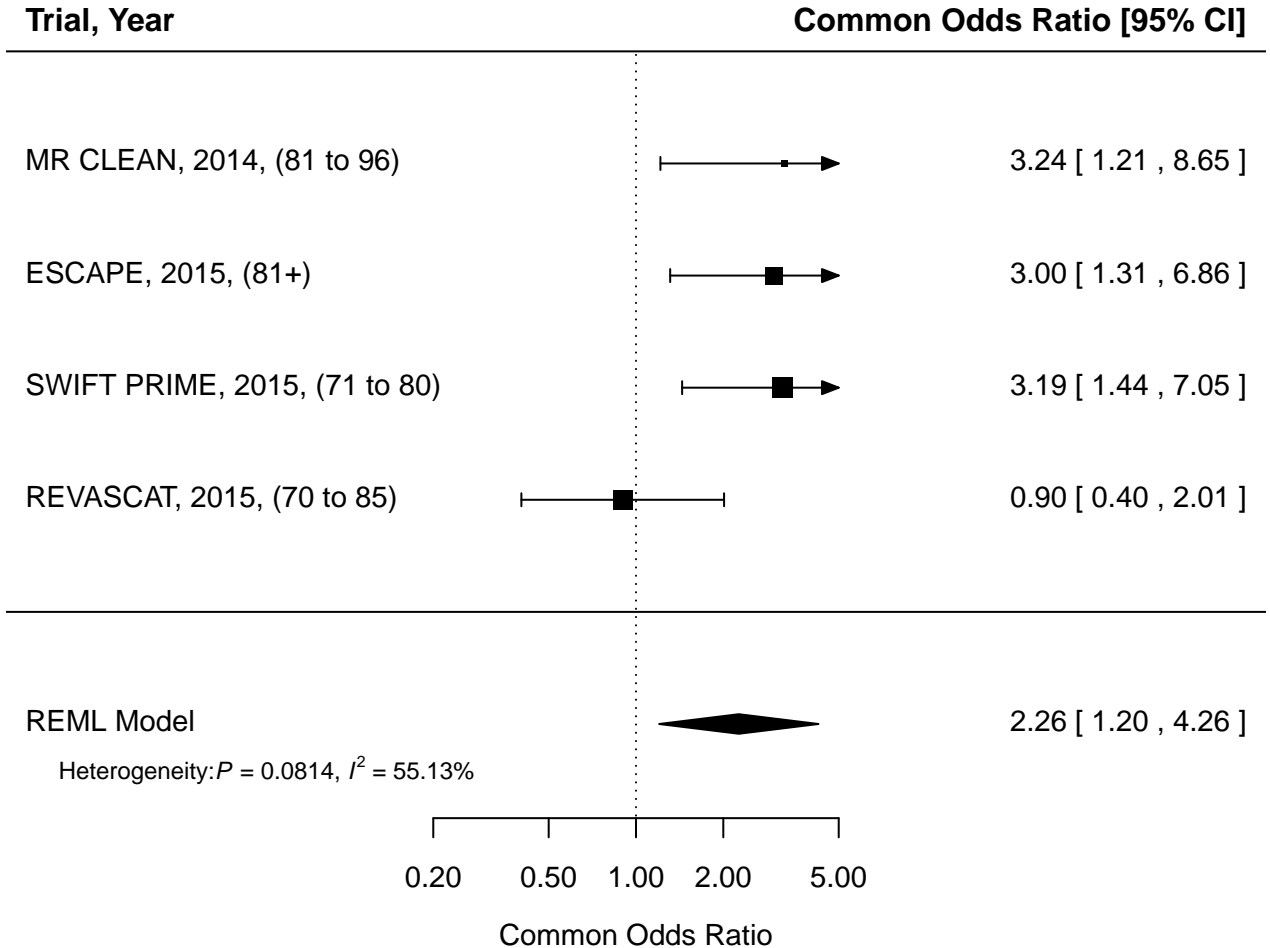

Supplement: S1 Data and Analyses — Datasets containing all abstracted data used in statistical analyses as well as R scripts to perform analyses presented are supplied in this package. (ZIP) [file pone.0147287.s001.zip › Data and Analyses/Subgroups/02.agegt70.pdf]

# Age less than 70

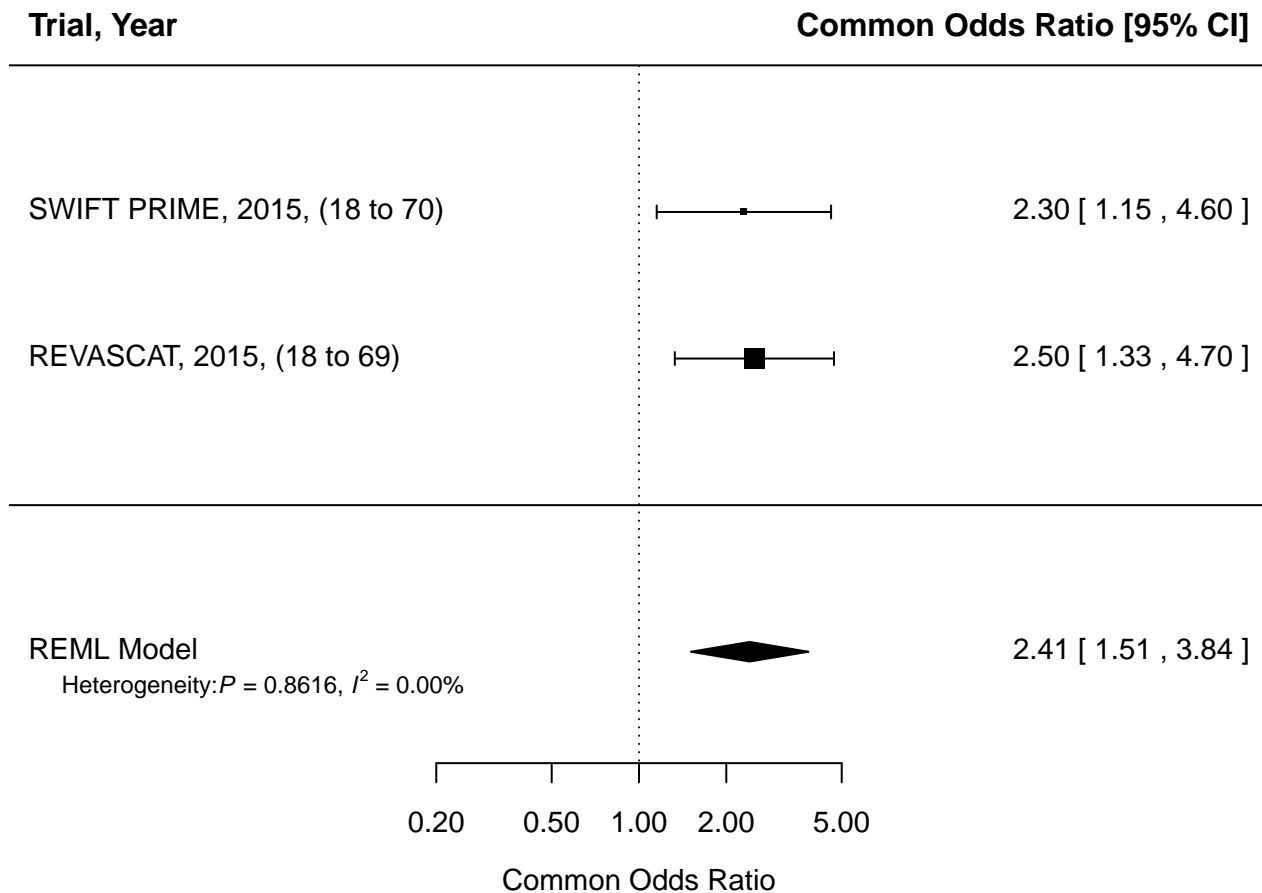

Supplement: S1 Data and Analyses — Datasets containing all abstracted data used in statistical analyses as well as R scripts to perform analyses presented are supplied in this package. (ZIP) [file pone.0147287.s001.zip › Data and Analyses/Subgroups/02.agelt70.pdf]

High Baseline NIHSS

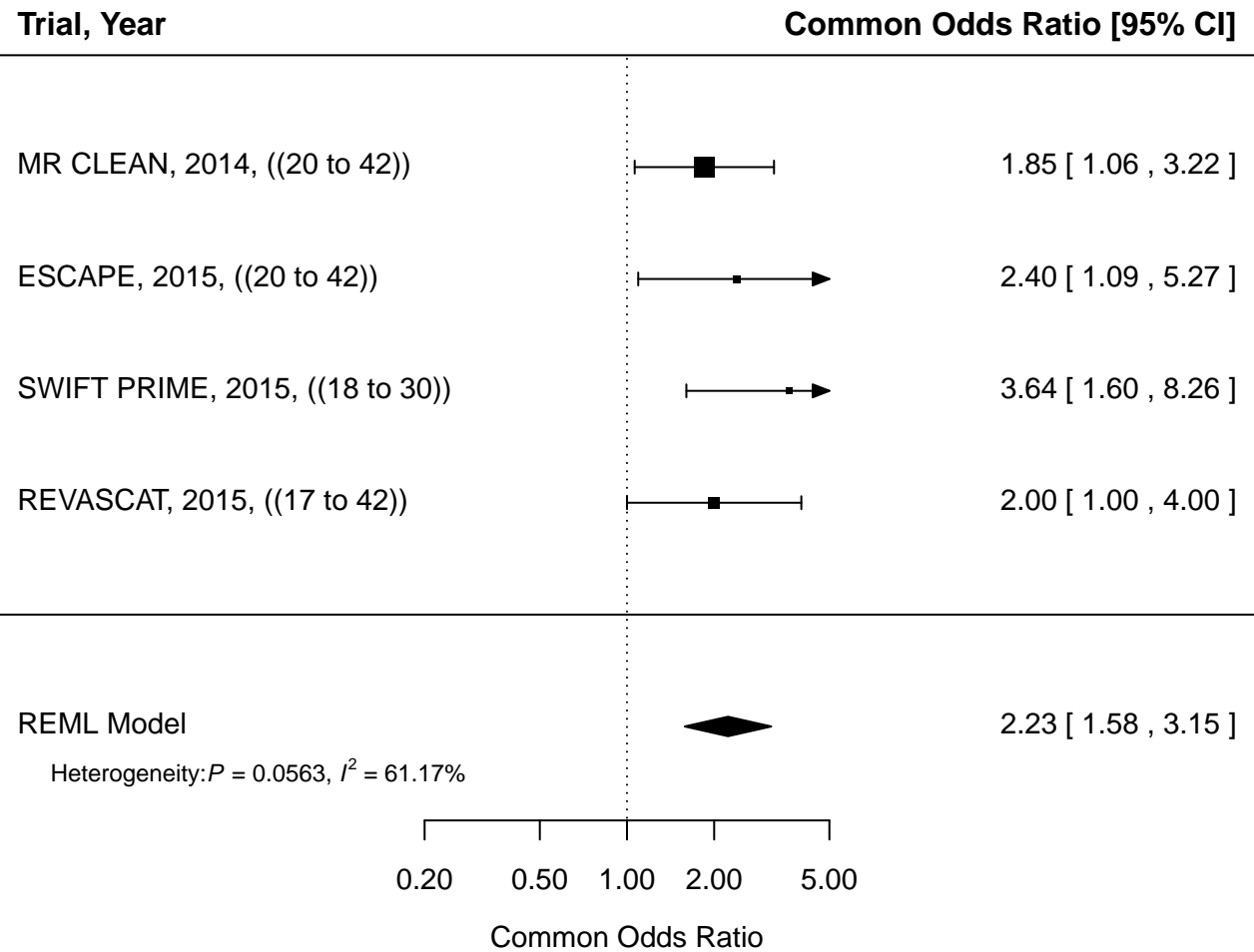

Supplement: S1 Data and Analyses — Datasets containing all abstracted data used in statistical analyses as well as R scripts to perform analyses presented are supplied in this package. (ZIP) [file pone.0147287.s001.zip › Data and Analyses/Subgroups/03.nihssgt17.pdf]

# Low Baseline NIHSS

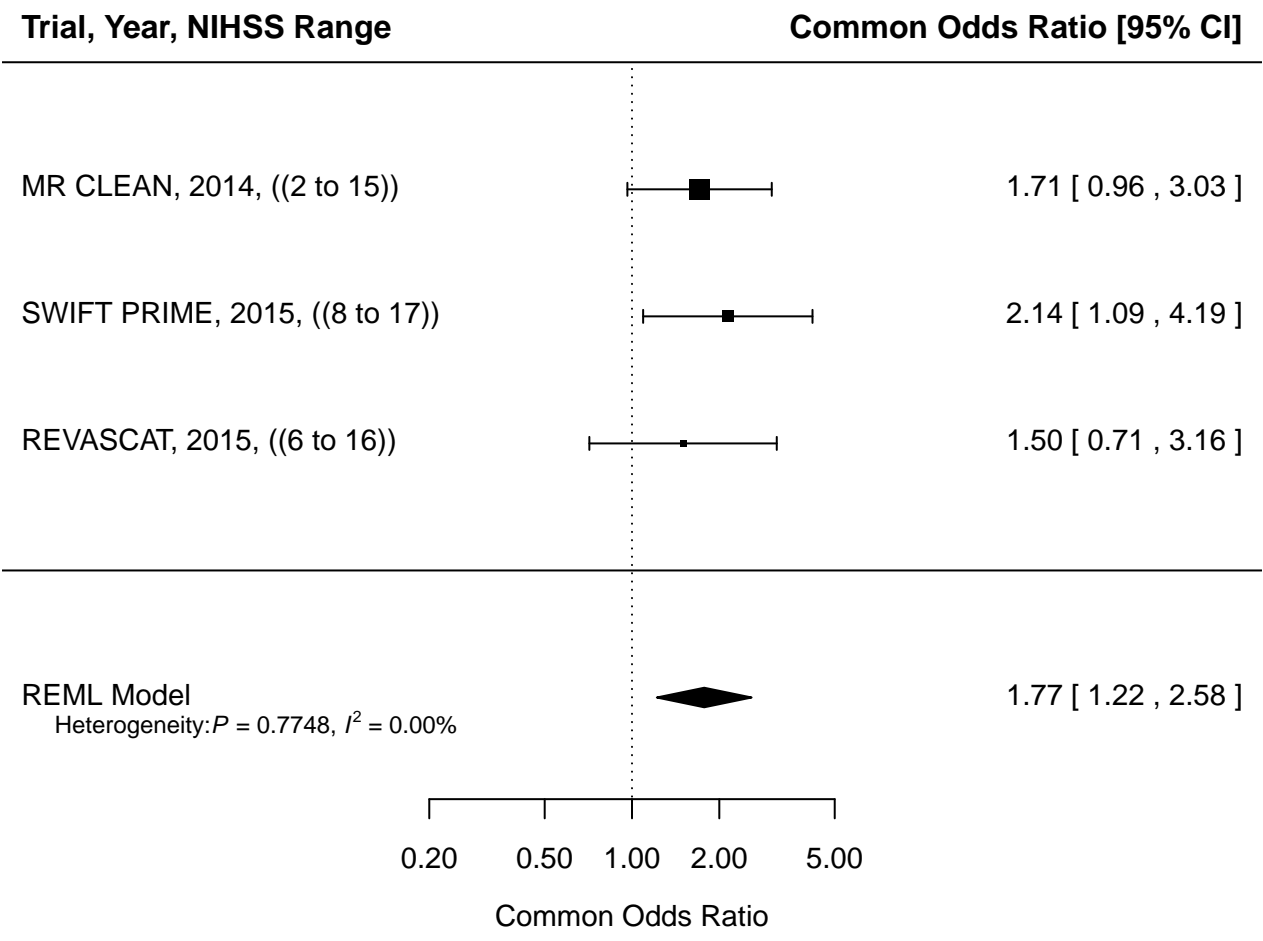

Supplement: S1 Data and Analyses — Datasets containing all abstracted data used in statistical analyses as well as R scripts to perform analyses presented are supplied in this package. (ZIP) [file pone.0147287.s001.zip › Data and Analyses/Subgroups/03.nihsslt17.pdf]

# ASPECTS $\geq 8$

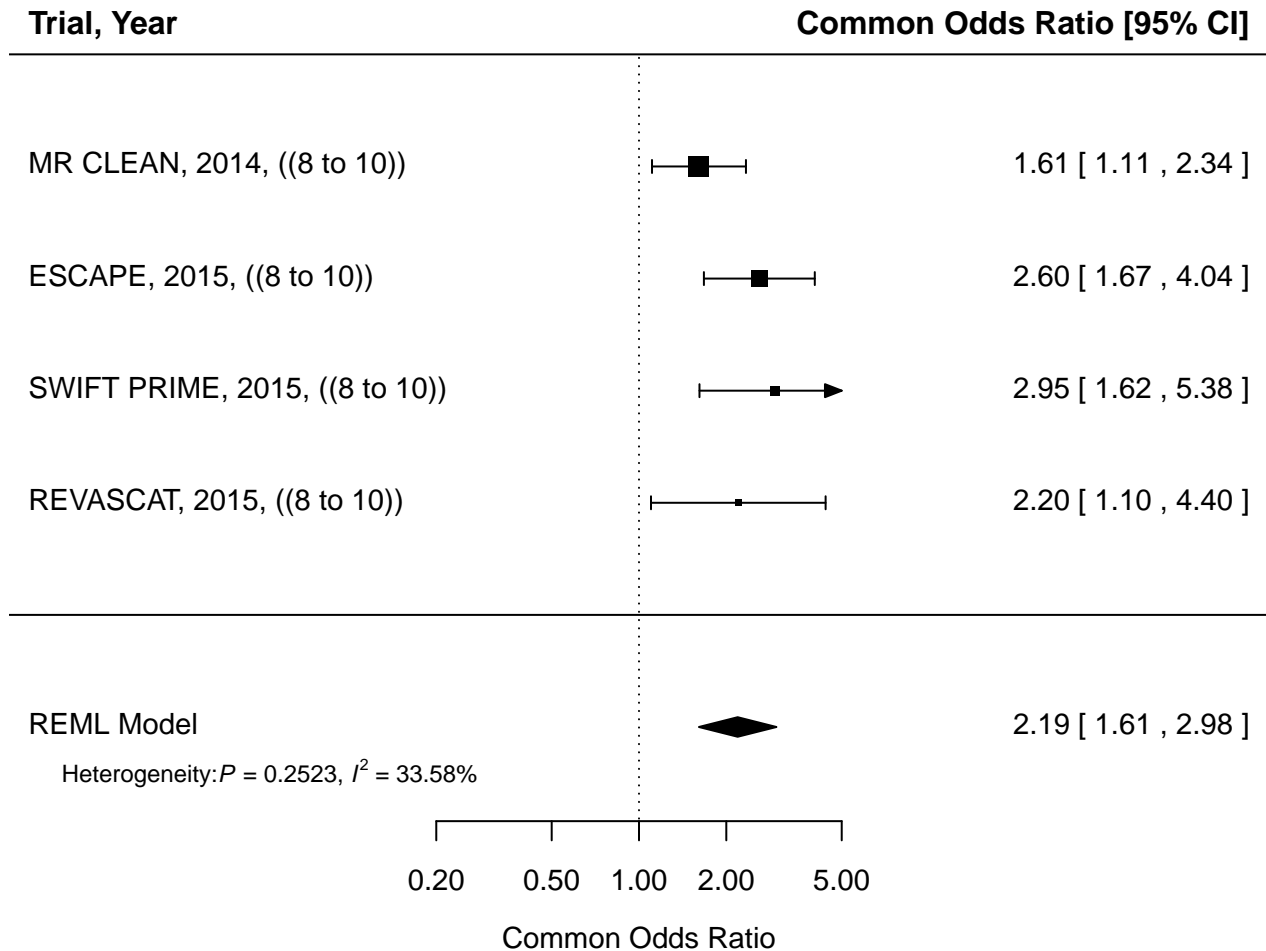

Supplement: S1 Data and Analyses — Datasets containing all abstracted data used in statistical analyses as well as R scripts to perform analyses presented are supplied in this package. (ZIP) [file pone.0147287.s001.zip › Data and Analyses/Subgroups/04.aspectshigh.pdf]

tPA Given

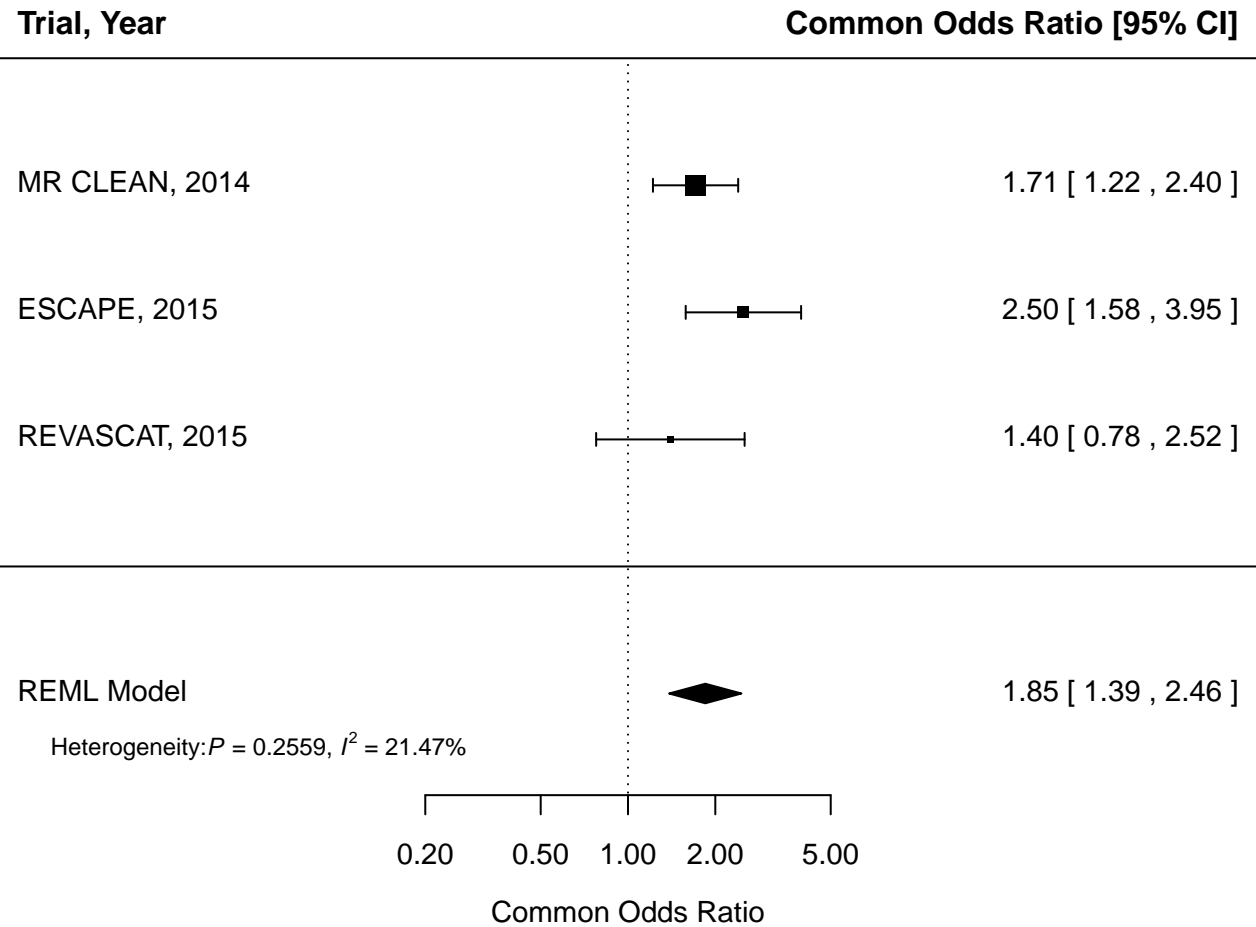

Supplement: S1 Data and Analyses — Datasets containing all abstracted data used in statistical analyses as well as R scripts to perform analyses presented are supplied in this package. (ZIP) [file pone.0147287.s001.zip › Data and Analyses/Subgroups/05.tpagiven.pdf]

# tPA Not Given

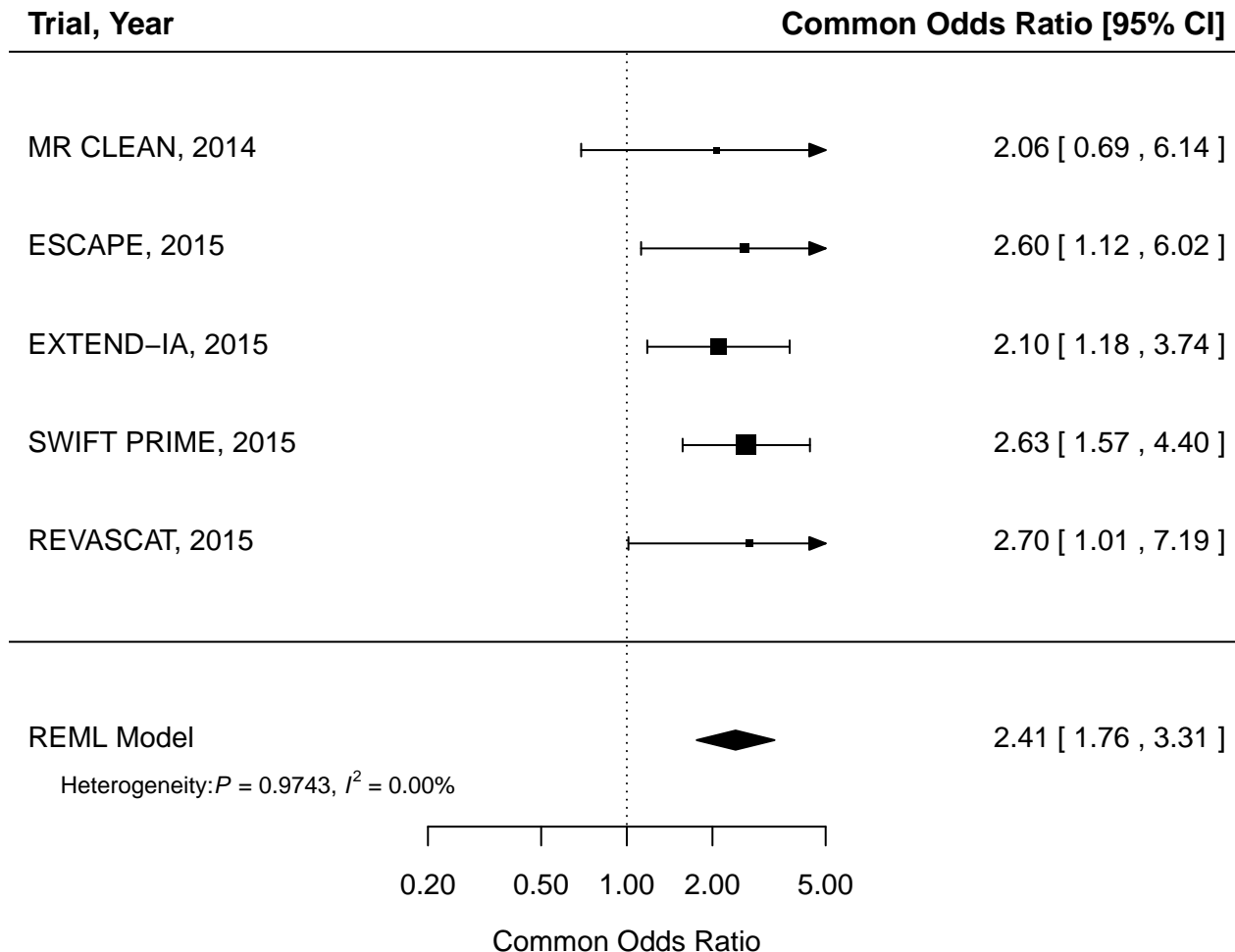

Supplement: S1 Data and Analyses — Datasets containing all abstracted data used in statistical analyses as well as R scripts to perform analyses presented are supplied in this package. (ZIP) [file pone.0147287.s001.zip › Data and Analyses/Subgroups/05.tpanotgiven.pdf]

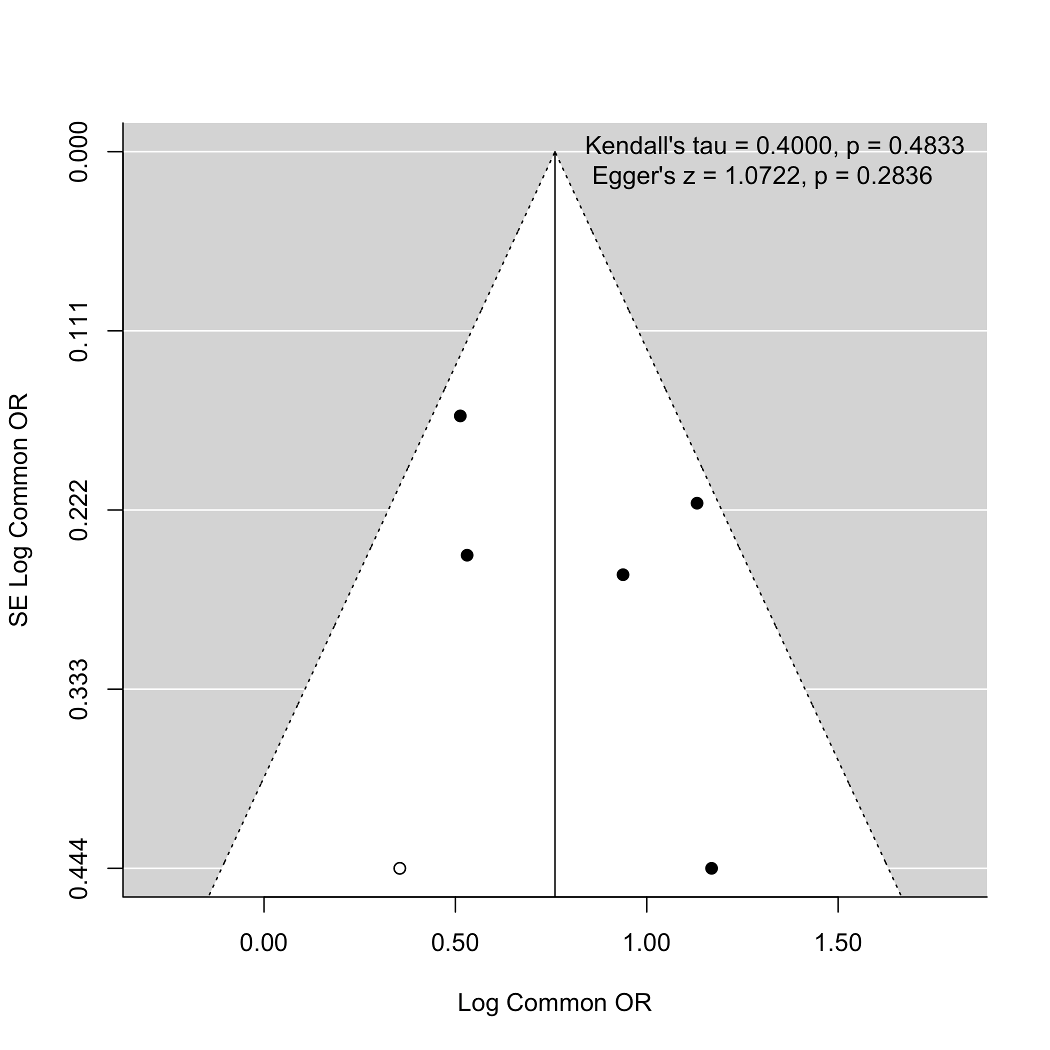

Supplement: S1 Fig — Both Kendall’s rank correlation and Egger’s mixed regression tests for missing studies were statistically nonsignificant. The single hollow point represents a non-significant result imputed with Duval and Tweedie’s trim and fill method. (TIF) [file pone.0147287.s002.tif]

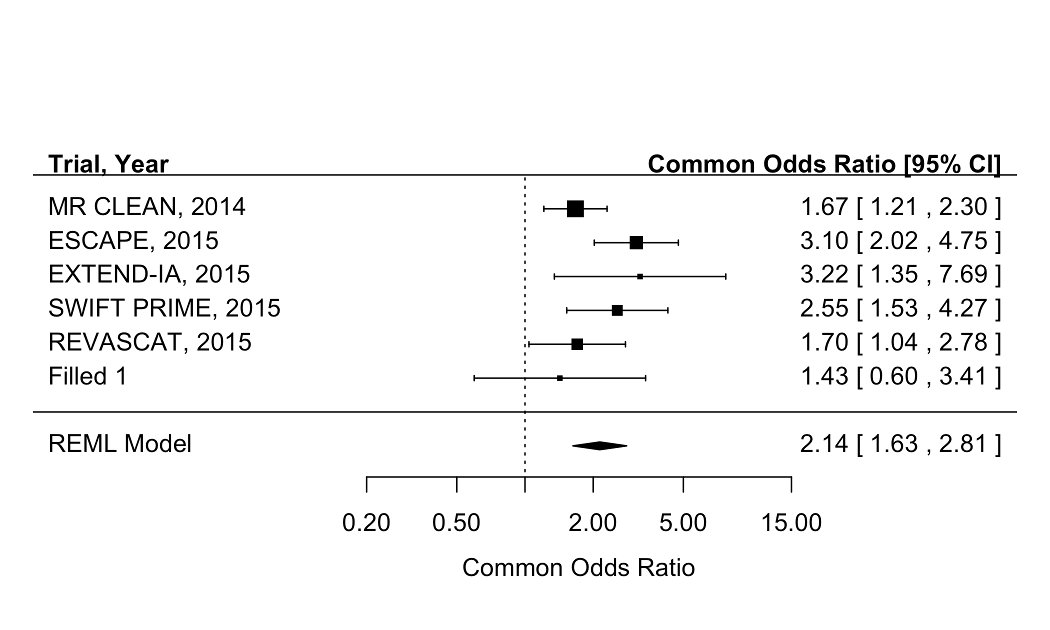

Supplement: S2 Fig — Duval and Tweedie’s trim and fill method for imputing missing study results provided a single, non-significant positive result, which has been pooled here with the other included trial results. (TIF) [file pone.0147287.s003.tif]

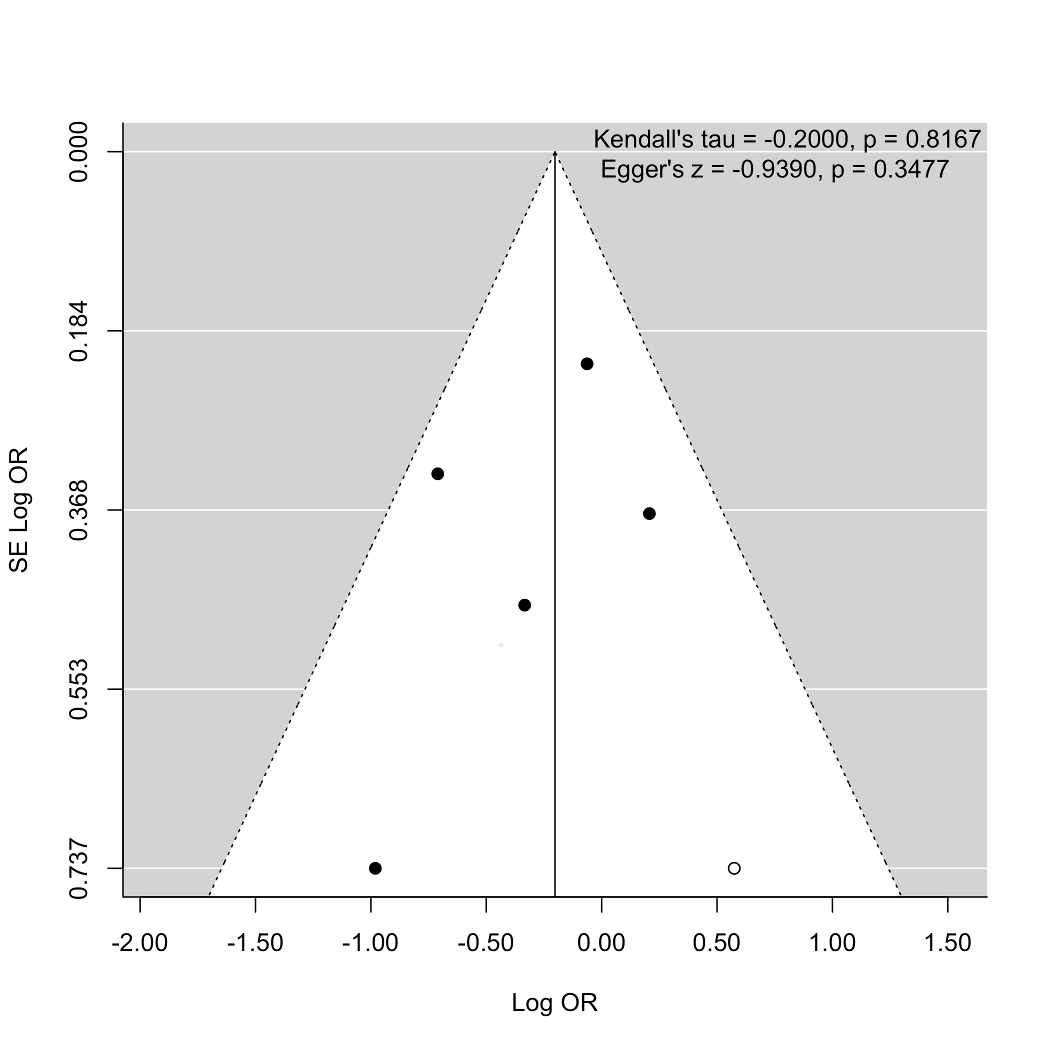

Supplement: S3 Fig — Both Kendall’s rank correlation and Egger’s mixed regression tests for missing studies were statistically nonsignificant. The single hollow point represents a non-significant result imputed with Duval and Tweedie’s trim and fill method. (TIF) [file pone.0147287.s004.tif]

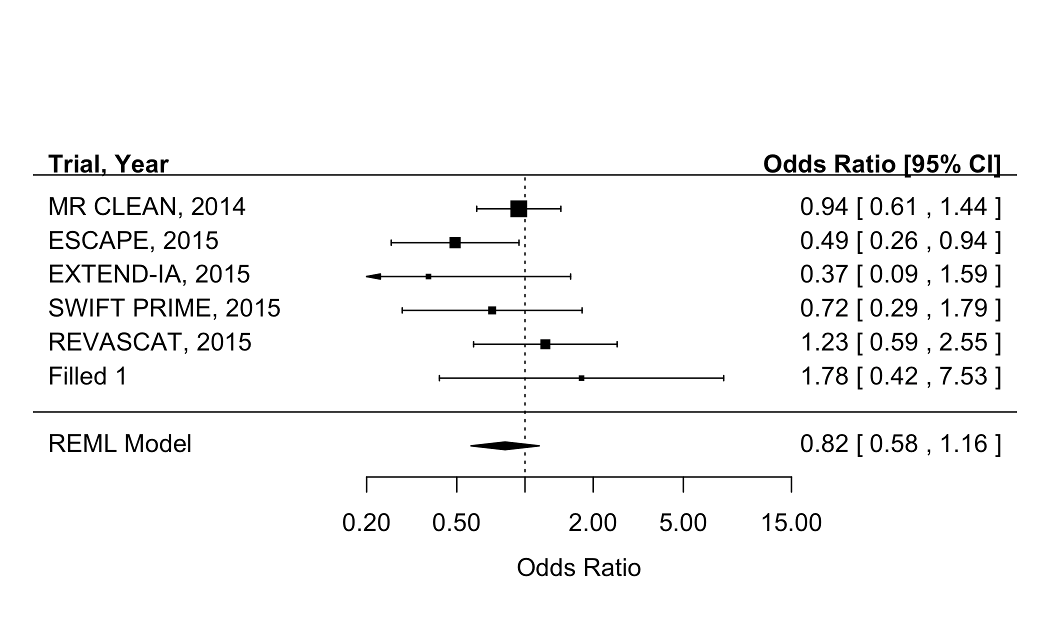

Supplement: S4 Fig — Duval and Tweedie’s trim and fill method for imputing missing study results provided a single, non-significant positive result, which has been pooled here with the other included trial results. (TIF) [file pone.0147287.s005.tif]

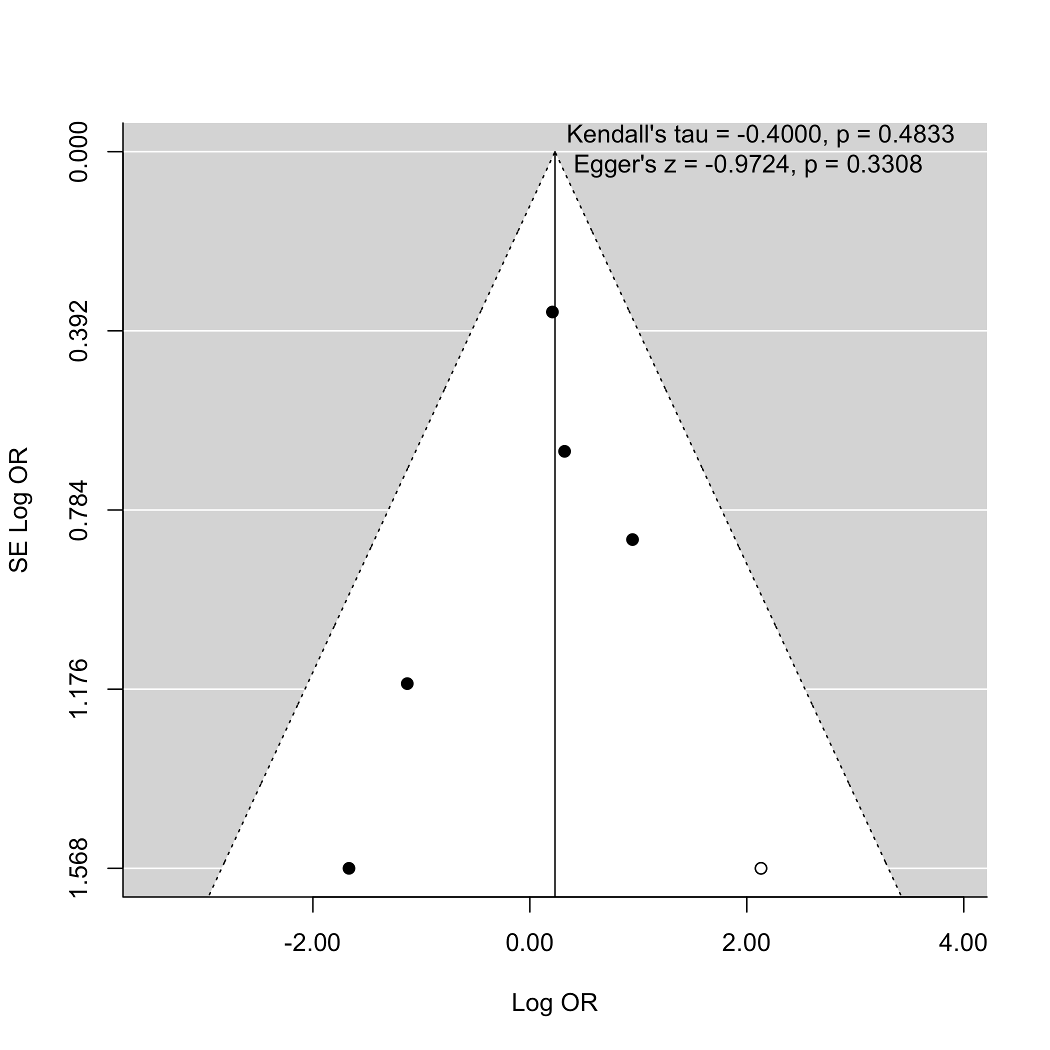

Supplement: S5 Fig — Both Kendall’s rank correlation and Egger’s mixed regression tests for missing studies were statistically nonsignificant. The single hollow point represents a non-significant result imputed with Duval and Tweedie’s trim and fill method. (TIF) [file pone.0147287.s006.tif]

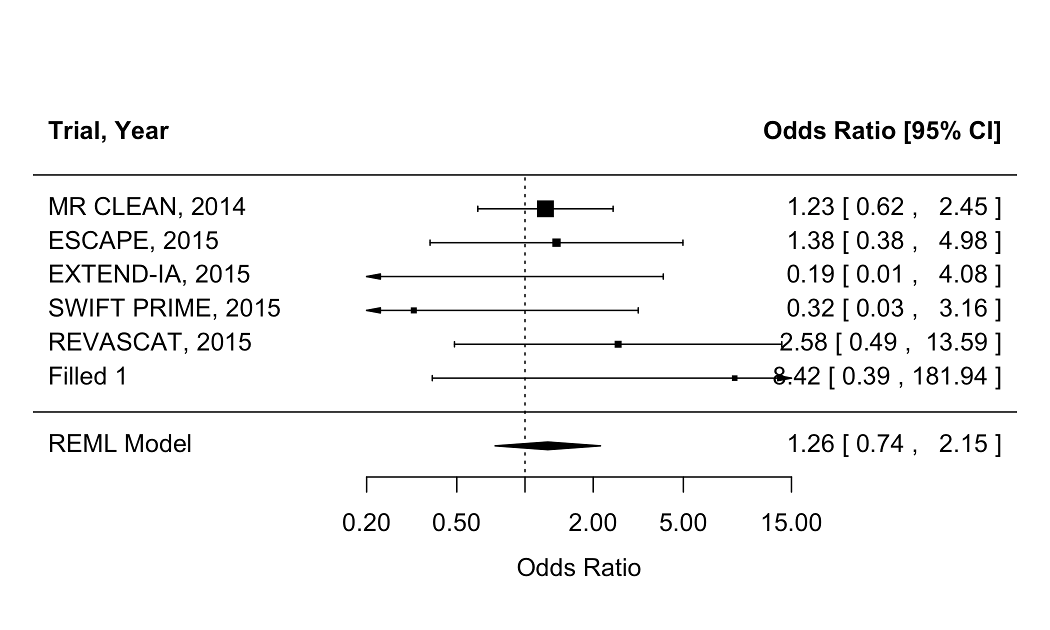

Supplement: S6 Fig — Duval and Tweedie’s trim and fill method for imputing missing study results provided a single, non-significant positive result, which has been pooled here with the other included trial results. (TIF) [file pone.0147287.s007.tif]
