# Supplementary material for: Endovascular Treatment with Stent-Retriever Devices for Acute Ischemic Stroke: A Meta-Analysis of Randomized Controlled Trials
Source: PLoS One. 2016 Jan 25;11(1):e0147287. doi: 10.1371/journal.pone.0147287 (PMC4726653; doi:10.1371/journal.pone.0147287)
Supplement: S1 Text — Exact search terms and syntax used to produce the initial search results. (DOCX) [file pone.0147287.s008.docx]

**Supplementary Methods**

***Search Strategy***

The following are exact searches with terms, logical structure, and limitations employed in the meta-analysis for each database searched. All searches were performed to include references up to November 27, 2015.

A. The following search was performed in MEDLINE. The items were combined as (1 AND 2 AND 3):

1. (("brain infarction"[MeSH Terms] OR ("brain"[All Fields] AND "infarction"[All Fields]) OR "brain infarction"[All Fields]) OR (("ischemia"[MeSH Terms] OR "ischemic"[All Fields]) AND ("stroke"[MeSH Terms] OR "stroke"[All Fields])))
2. ((("endovascular"[All Fields] AND ("therapy"[All Fields] OR "treatment"[All Fields] OR "intervention"[All Fields])) OR "endovascular procedures"[MeSH Terms]) OR (("intravenous"[All Fields] AND "thrombolysis"[All Fields]) OR ("tissue plasminogen activator"[MeSH Terms] OR ("tissue"[All Fields] AND "plasminogen"[All Fields] AND "activator"[All Fields]) OR "tissue plasminogen activator"[All Fields] OR ("recombinant"[All Fields] AND "tissue"[All Fields] AND "plasminogen"[All Fields] AND "activator"[All Fields]) OR "recombinant tissue plasminogen activator"[All Fields])))
3. (("humans"[MeSH Terms]) AND ("adult"[MeSH Terms]) AND ("Randomized Controlled Trial"[ptyp]))

B. The following search was performed in Embase. The items were combined as (((1 OR 2) AND 3) AND 4 AND 5 AND 6 AND 7 AND 8):

1. 'endovascular treatment' OR ('endovascular' AND 'therapy') OR 'intra-arterial'
2. 'intravenous'/exp OR 'intravenous'
3. 'fibrinolysis'/exp OR 'fibrinolysis' OR 'thrombolysis'/exp OR 'thrombolysis'
4. 'ischemic stroke'/exp OR 'ischemic stroke' OR 'brain infarct'/exp OR 'brain infarct' OR 'brain ischemia'/exp OR 'cerebrovascular accident'/exp
5. [randomized controlled trial]/lim
6. ([adult]/lim OR [aged]/lim)
7. [humans]/lim
8. [embase]/lim

C. The following search was performed in Cochrane Central Register of Controlled Trials. The items were combined as (1 AND 2 AND 3):

1. (((endovascular therapy):ti,ab,kw or (endovascular surgery):ti,ab,kw) or (tissue plasminogen activator):ti,ab,kw)
2. ((ischemic stroke):ti,ab,kw or (brain ischemia):ti,ab,kw or (cerebrovascular accident):ti,ab,kw)
3. (randomized):pt

D. The following search was performed in Web of Science. The items were combined as (1 AND (2 OR 3) AND 4):

1. TS=((brain AND (isch*emia OR infarct)) OR (ischemic stroke) OR (cerebrovascular AND accident))
2. TS=(endovascular AND (treatment OR therapy OR intervention))
3. TS=(((intra*arterial OR intravenous) AND (thrombolysis OR thrombolytic OR fibrinolysis OR fibrinolytic)) OR (tissue plasminogen activator OR alteplase OR duteplase OR monteplase OR silteplase))
4. TS=(randomized controlled trial)

E. The following search was performed in National Institutes of Health ClinicalTrials.gov:

("stroke") AND ("endovascular procedures" OR "tissue plasminogen activator") | Interventional Studies
