# Supplementary material for: Endovascular Treatment with Stent-Retriever Devices for Acute Ischemic Stroke: A Meta-Analysis of Randomized Controlled Trials
Source: PLoS One. 2016 Jan 25;11(1):e0147287. doi: 10.1371/journal.pone.0147287 (PMC4726653; doi:10.1371/journal.pone.0147287)
Supplement: S2 Text — The project proposal and a priori protocol is provided. (DOCX) [file pone.0147287.s009.docx]

Endovascular Treatment for Acute Ischemic Stroke:

A Meta-Analysis of Randomized Controlled Trials

Proposal and Protocol

March 2, 2015

Chad K. Bush, BS^1,2^; Lee J. Cross, MPH^1^; Dayaamayi Kurimella, BFA^1^; Katherine Conner, MPH^1^; and Tanika Kelly, PhD^1^

^1^ Department of Epidemiology, Tulane University School of Public Health and Tropical Medicine, New Orleans, Louisiana, USA

^2^ Department of Medicine, Tulane University School of Medicine, New Orleans, Louisiana, USA

**Introduction**

Stroke was the second leading cause of death worldwide in 2010 (Lozano, Naghavi, Foreman, Lim, Shibuya, & al, 2012) and is a leading cause of long-term disability in the US (Centers for Disease Control and Prevention (CDC), 2009). In the US, 87% of all strokes are ischemic (Mozaffarian, et al., 2015), caused by occlusion of an intracerebral artery by thrombus or embolism. The standard treatment for acute ischemic stroke is delivery of an intravenous thrombolytic agent, recombinant tissue plasminogen activator (t-PA) (The National Institute of Neurological Disorders and Stroke rt-PA Stroke Study Group, 1995). Thrombolytic stroke therapy is based on the recanalization hypothesis, i.e., that clinical outcome after acute ischemic stroke is dependent upon reopening occluded vessels, resulting in reperfusion and salvage of affected brain tissue. It has been shown that t-PA is clinically effective only within a narrow window of 3 to 4.5 hours after onset of stroke symptoms (Lees, et al., 2010), after which the likelihood of neurological and functional recovery decreases markedly. Because of the short therapeutic window for IV t-PA, and because of the extensive set of other clinical eligibility criteria for administration, limited acute ischemic stroke patients qualify for the intervention on presentation.

Endovascular treatment, involving minimally invasive techniques for thrombolysis, thrombectomy, thrombus retrieval and / or stent placement, has been explored as an alternative or adjunct to IV t-PA for many years (Broderick, et al., 2013). When compared to control group, endovascular treatment is associated with a higher probability of recanalization (Furlan, et al., 1999) (Lee, Hong, & Saver, 2010). Results of trials of endovascular treatment compared with IV t-PA, though, have been varied, with most early trials showing no significant treatment differences (Singh, Parsaik, Prokop, & Mittal, 2013). However, more recent trials have demonstrated significant benefits for endovascular treatment in ischemic stroke (Berkhemer, et al., 2015) (Campbell, et al., 2015) (Goyal, et al., 2015) (Saver, et al., 2015) (Jovin, et al., 2015). A meta-analysis of randomized controlled trials (RCTs) examining the effects of endovascular treatment on ischemic stroke is needed to provide a more precise estimate of treatment effects and explain the inconsistency in past findings.

The aim of our study is to perform a comprehensive systematic review and meta-analysis of all published RCTs to compare the efficacy of endovascular treatment with or without IV t-PA with standard of care in patients with acute ischemic stroke (AIS). Our primary outcomes will be modified Rankin Scale score (mRS), a measure of overall patient disability, of 0 to 2 out of 6 (so-called “functional independence”) and all-cause mortality. Our secondary safety outcome of symptomatic intracerebral hemorrhage (sICH) will be ascertained and compared as well. We plan to perform subgroup analyses for endovascular treatment versus IV t-PA alone and specific ET procedure, e.g., intra-arterial thrombolysis versus endovascular thrombectomy with stent retriever system.

**Methods**

***Eligibility Criteria:***

The studies included in this meta-analysis are RCTs of ET compared to standard of care for AIS patients. Studies will be included in this meta-analysis based on the following criteria:

1. ET treatment is part of the intervention.
2. Standard of care is provided to the comparison group.
3. Specific aspects of standard of care are clearly defined among comparison groups, e.g.,  aspirin, heparin, other antiplatelet or anticoagulant agents, recombinant tissue  plasminogen activator, alteplase, urokinase, etc.
4. Treatment allocation is random.
5. At least one of the following:
6. Primary outcome is number of patients attaining 90-day functional independence, as measured via the modified Rankin Scale (mRS) score of 0-2.
7. Secondary outcomes
   1. 90-day all-cause mortality for each treatment arm.
   2. Risk of symptomatic intracerebral hemorrhage (sICH) for each treatment arm
8. A risk estimate is provided, e.g., relative risk, risk ratio, rate ratio, odds ratio, or data from which a risk estimate can be calculated for outcome measures.
9. Measures of variance, confidence intervals and/or p-values are reported or data from which they can be calculated are provided.
10. Study participants are at least 18 years of age.
11. No differences between comparison groups, other than specified intervention allocation,  exist.
12. Duration of follow-up is 90 days.
13. Study is not a duplicate publication of a trial already included in meta-analysis, or if study is a duplicate publication, it represents the most recent and/or the most complete publication of information from that trial.

***Information Sources***

Studies will be retrieved by systematically searching MEDLINE, EMBASE, the Cochrane library, and Web of Science, without language restriction, for RCTs that meet eligibility criteria. A manual search of references from all articles meeting eligibility, along with relevant review articles, systematic reviews and meta-analyses, will also be conducted. Recent studies will be sought to add to the previous body of knowledge. Study authors will not be contacted to identify additional studies.

***Search Strategy***

Two investigators will independently search the MEDLINE database from Januay 1, 1950 up to May 2015, for the following terms as Medical Subject Headings (MeSH) and keywords: “ischemic stroke”, “brain infarct”, “endovascular treatment”, “intra-arterial”, “intra-venous”, “fibrinolysis”, and “thrombolysis” (see Appendix A for further details). The search will be restricted to include only randomized controlled trials conducted among humans, and no language restrictions will be used.

***Study Selection Criteria and Procedures***

Two investigators will independently and in duplicate screen the abstract and titles of articles generated by the literature search using the MeSH terms and keywords previously specified. The titles and abstracts will be reviewed to identify articles that meet the previously stated inclusion criteria. Articles that appear to meet inclusion criteria will be retrieved, and any articles that are deemed ineligible based on title and abstract review will be excluded. Retrieved articles will then be reviewed in full text. The selection results from each researcher’s review will be compared to ensure that all relevant articles have been retrieved. A third investigator will make final decisions on any discrepancies in selected articles between the two reviews. Only the most complete and up-to-date results of studies that are published more than once will be included in the analysis.

***Data Abstraction***

Data will be abstracted from included studies by two independent investigators. Each investigator will use a standardized collection form (see Appendix B). The results of the duplicate data abstraction will be compared, and discrepancies in data collection between data abstracted by each researcher will be resolved by discussion and consensus. The following data will be abstracted from each study: citation information (e.g., title, first author’s name, and publication year), type of study design, randomization method, randomization adequacy, blinding method, blinding adequacy, sample size, participant characteristics (age, gender, race/ethnicity), met inclusion criteria, treatment sub-type (intra-arterial thrombolysis, endovascular thrombectomy, or other ET modalities), control arm medical management subtype (IV-tPA only, IV-tPA if candidate, IV anticoagulation, other medical interventions), mean or median time-to-intervention in minutes, study duration and duration of follow-up, response rate (e.g., withdrawals and dropouts), and adherence to intention-to-treat (ITT) principle.

***Outcome Measures***

The primary prespecified outcome measure collected will be the odds ratio of patients achieving a modified Rankin Scale (mRS) score of 0-2 at 90 days for each treatment arm. The mRS score is universally accepted and reported as an outcome measure among all major stroke trials. mRS scores range from a score of 0, indicating full functional independence without symptoms or deficits, to a score of 6, indicating death. The prespecified outcome of mRS of 0-2 at 90 days is also referred to as an outcome of functional independence. The other pre-specified primary outcome will be all-cause mortality at 90 days. The pre-specified secondary safety outcome will be symptomatic intracerebral hemorrhage for control and treatment arms. For secondary outcomes, number of events and participants in each arm as well as reported measures of association will be collected, with standard deviations, confidence intervals, and/or p-values.

***Synthesis of Results***

Heterogeneity of treatment effects between studies will be statistically examined using Cochran’s Q test statistic and the associated I2 heterogeneity estimate, with an α level prespecified at 0.10, and an influence analysis of heterogeneous effects on pooled outcomes will be conducted. The pre-specified method for pooled effects analysis will be an inverse-variance weighted restricted maximum-likelihood (REML) random-effects model. REML was chosen *a priori* since the method is highly robust to underlying violations of assumptions that cause other estimators to yield unstable pooled estimates (Viechtbauer, 2005), and heterogeneity is expected to be significant given variations in RCT sample sizes and global populations along with significant advances in interventional technologies over the decades during which ET for AIS trials have been conducted. Statistical analyses will be conducted in R 3.1.3. The meta-analysis will be performed in accordance with the Preferred Reporting Items for Systematic Reviews and Meta-Analyses (PRISMA) guidelines (Moher, Liberati, Tetzlaff, Altman, & Group, 2009).

***Assessment of Study Quality***

An assessment of study quality will be performed at the time of analysis. The standardized 5-point Jadad scale will be used to assess various aspects of study quality (Jadad, et al., 1996). Study aspects to be evaluated are: whether randomization occurred, whether the randomization method was described and appropriate, whether blinding occurred, whether the method of blinding was described and appropriate, and whether a description of withdrawals and dropouts was provided. In departure from the original scales proposed by Jadad, et al., 2006, RCTs of ET for AIS were considered to have been doubly blinded if both medical providers performing follow-up examinations were unaware of patients’ intervention-arm randomization and allocation and statistical analyses were performed by statisticians blinded to patient treatment. Studies with a total Jadad score of 3 or more were considered high quality while studies with score of 2 or less were considered low quality.

***Risk of Bias Across Studies and Additional Analyses***

Publication bias will be assessed using visual inspection of funnel plots on which standard errors will be plotted against effect sizes. Both Kendall’s rank correlation statistic and Egger’s mixed regression tests for funnel plot asymmetry will be calculated to assess for statistically significant funnel plot asymmetry. Duval and Tweedie’s nonparametric trim-and-fill method will be employed to detect and estimate effects of missing studies. Sensitivity and influence analyses will be performed assessing impact of heterogeneity of trial results on pooled effects estimates. Sub-group analyses of trials comparing ET with or without IV-tPA to IV-tPA alone will be planned and performed. Meta-regression of study effects against mean or median study time-to-intervention in minutes will be performed.

**References**

Broderick, J., Palesch, Y., Demchuk, A., Yeatts, S., Khatri, P., Hill, M., et al. (2013). Endovascular therapy after intravenous tPA versus tPA alone for stroke. *N Engl J Med* *, 368* (10), 893-903.

Furlan, A., Higashida, R., Wechsler, L., Gent, M., Rowley, H., Kase, C., et al. (1999). Intra-arterial prourokinase for acute ischemic stroke. The PROACT II student: a randomized controlled trial. *JAMA* *, 282* (21), 2003-2011.

Goyal, M., Demchuck, A., Menon, B., Eesa, M., Rempel, J., Thornton, J., et al. (2015). Randomized assessment of rapid endovascular treatment of ischemic stroke. *N Engl J Med* .

Jadad, A., Moore, R., Carroll, D., Jenkinson, C., Reynolds, D., Gavaghan, D., et al. (1996). Assessing the quality of reports of randomized clinical trials: is blinding necessary? *Controlled Clinical Trials* *, 17*, 1-12.

Lee, M., Hong, K., & Saver, J. (2010). Efficacy of intra-arterial fibrinolysis for acute ischemic stroke: meta-analysis of randomized controlled trials. *Stroke* *, 41*, 932-937.

Lees, K., Bluhmki, E., von Kummer, R., Brott, T., Toni, D., Grotta, J., et al. (2010). Time to treatment with intravenous alteplase and outcome in stroke: an updated pooled analysis of ECASS, ATLANTIS, NINDS, and EPITHET trials. *Lancet* *, 375*, 1695-1703.

Lloyd-Jones, D., Adams, R., Brown, T., Carnethon, M., Dai, S., De Simone, G., et al. (2010). Executive Summary: Heart Disease and Stroke Statistics - 2010 Update: A Report from the American Heart Association. *Circulation* *, 121*, 948-954.

Moher, D., Liberati, A., Tetzlaff, J., Altman, D.G., PRISMA Group (2009). Preferred Reporting Items

for Systematic Reviews and Meta-Analyses: The PRISMA Statement. *Annals of Internal Medicine, 151,* 264-269.

Singh, B., Parsaik, A., Prokop, L., & Mittal, M. (2013). Endovascular therapy for acute ischemic stroke: a systematic review and meta-analysis. *Mayo Clin Proc* *, 88* (10), 1056-1065.

The National Institute of Neurological Disorders and Stroke rt-PA Stroke Study Group. (1995). Tissue Plasminogen Activator for Acute Ischemic Stroke. *N Engl J Med* *, 333* (24), 1581-1587.

**Appendix A: Search Terms**

The following search terms will be used to identify relevant articles in MEDLINE. These terms will be combined as ((1 AND (2 OR 3)) AND 4 AND 5 AND 6):

1. (“brain infarction”[MeSH Terms] OR (“brain”[All Fields] AND “infarction”[All Fields]) OR “brain infarction”[All Fields]) OR ((“ischemia”[MeSH Terms] OR “ischemic”[All Fields]) AND (“stroke”[MeSH Terms] OR “stroke”[All Fields])
2. (endovascular[All Fields] AND ("therapy"[Subheading] OR "therapy"[All Fields] OR "treatment"[All Fields] OR "therapeutics"[MeSH Terms] OR "therapeutics"[All Fields]))
3. (intravenous[All Fields] AND thrombolysis[All Fields]) OR ("tissue plasminogen activator"[MeSH Terms] OR ("tissue"[All Fields] AND "plasminogen"[All Fields] AND "activator"[All Fields]) OR "tissue plasminogen activator"[All Fields] OR ("recombinant"[All Fields] AND "tissue"[All Fields] AND "plasminogen"[All Fields] AND "activator"[All Fields]) OR "recombinant tissue plasminogen activator"[All Fields])
4. “humans”[MeSH Terms]
5. “adult”[MeSH Terms]
6. “Randomized Controlled Trial”[ptyp]

The following terms will be used to identify relevant articles in EMBASE:

1. “ischemic stroke”/exp
2. “endovascular therapy”/exp
3. “intravenous thrombolysis”/exp
4. “tissue plasminogen activator”/exp
5. [adult]/lim OR [aged]/lim
6. [humans]/lim
7. [randomized controlled trial]/lim
8. [embase]/lim

The following search terms will be used in the Cochrane library:

1. (ischemic stroke):ti,ab,kw
2. (endovascular therapy):ti,ab,kw
3. (intravenous thrombolysis):ti,ab,kw
4. (tissue plasminogen activator):ti,ab,kw
5. (randomized):pt

The following search terms will be used to identify relevant articles in Web of Science:

1. Topic=(randomized controlled trial)
2. Topic=(ischemic stroke)
3. Topic=(endovascular therapy)
4. Topic=(intravenous thrombolysis)
5. Topic=(tissue plasminogen activator)

**Appendix B – Data Abstraction Form**

Study ID number

Date of Abstraction: / / 2015

Initials of Data Abstractor:

**General Information**

1. Article title

1. Authors (last name, first initial)

1. Publication year:
2. Journal title:
3. Journal volume number:
4. Issue number:
5. Page numbers:
6. Study name (include acronym, if applicable):
7. Study country:

☐ U.S.

☐ Other(s) - specify:

**Study Characteristics**

1. Study design

☐ Randomized Clinical Trial

☐ NOT Randomized Clinical Trial (exclude)

1. Type of intervention:

☐ Endovascular thrombolysis (ET) (enter type of ET – as applicable):

☐ Intra-arterial thrombolysis

☐ Endovascular thrombectomy without retrievable stent

☐ Endovascular thrombectomy with retrievable stent

☐ Other, specify:

☐ Other, not ET, specify:

If not ET, then exclude.

1. Type of comparison (control) group:

☐ Standard of care / usual care (enter type):

☐ IV-tPA alone

☐ IV-tPA if candidates

☐ IV anticoagulation, specify:

☐ Other, specify:

If not usual care, then exclude.

1. Risk estimate for Modified Rankin Scale (mRS) score of functional independence (mRS ≤ 2) reported? (primary outcome)

☐ Yes, specify type:

☐ OR

☐ RR

☐ Other, specify:

☐ No

1. Measure of association for all-cause mortality reported or data from which it can be calculated? (secondary outcome)

☐ Yes

☐ RR

☐ OR

☐ Other, specify:

☐ No

1. Measure of association for symptomatic intracerebral hemorrhage (sICH) reported? (secondary outcome)

☐ Yes

☐ RR

☐ OR

☐ Other, specify:

☐ No

1. Is at least 1 of questions 13, 14, and 15 answered “yes”?

- Yes
- No *(exclude study; at least one of the outcome measures must be present)*

1. Length of follow up: (if not at equivalent to at least 90 days, then exclude)

- Days
- Weeks
- Months
- Years

1. Was randomization described and appropriate?

□ Described and appropriate

□ Described but was not appropriate

□ Not described so unknown whether appropriate

1. Why was randomization appropriate or not? ___________________________

_______________________________________________________________

________________________________________________________________

________________________________________________________________

1. Intention-to-treat analysis performed?

- Yes
- No

1. Blinding:

- Open
- Single (participant)
- Single (investigator or clinician)
- Single (analyst)
- Double (participant, investigator)
- Double (investigator or clinician, analyst)
- Triple (participant, investigator, analyst)

**Participant Characteristics**

| **Baseline** | **Control** | **Treatment** | **Comments** |
| --- | --- | --- | --- |
| N at start |  |  |  |
| Average age |  |  |  |
| Standard deviation |  |  |  |
| Sex |  |  |  |
| Male – n, % |  |  |  |
| Female – n, % |  |  |  |
| Race/ethnicity |  |  |  |
| White – n, % |  |  |  |
| Black – n, % |  |  |  |
| Asian – n, % |  |  |  |
| Hispanic – n, % |  |  |  |
| Other – n, % |  |  |  |
| Comorbidities |  |  |  |

(go to next page)

**Study Outcomes**

|  | **Control** | **Treatment** | **Comments** |
| --- | --- | --- | --- |
| **Compliance - %** |  |  |  |
| **Drop-out rate - %** |  |  |  |
| Missing - % |  |  |  |
| Loss to follow-up - % |  |  |  |
| **Functional Independence by 90-day modified Rankin Scale (mRS) score ≤ 2** | | | |
| N, % |  |  |  |
| OR, RR, HR (circle one) |  | |  |
| ­ % CI or SD/SE (circle one) |  | |  |
| p-value |  | |  |
| **90-day All-cause mortality** | | | |
| N, % |  |  |  |
| OR, RR, HR (circle one) |  | |  |
| ­ % CI or SD/SE (circle one) |  | |  |
| p-value |  | |  |
| **90-day Symptomatic Intracerebral Hemorrhage** | | | |
| N, % |  |  |  |
| OR, RR, HR (circle one) |  | |  |
| ­ % CI or SD/SE (circle one) |  | |  |
| p-value |  | |  |
| **Time Sxs-Onset-to-Treatment (min)** | | | |
| Mean, median (circle one) |  |  |  |
| % CI or SD/SE (circle one) |  |  |  |
